# Compartment-specific transcriptome of motor neurons reveals impaired extracellular matrix signaling and activated cell cycle kinases in FUS-ALS

**DOI:** 10.1016/j.nbd.2026.107268

**Published:** 2026-01-10

**Authors:** Vitaly Zimyanin, Banaja P. Dash, Theresa Simolka, Hannes Glaß, Arun Pal, Felix Haidle, Kathi Zarnack, Riya Verma, Vivek Khatri, Christopher Deppmann, Eli Zunder, Michaela Müller-McNicoll, Stefanie Redemann, Andreas Hermann

**Affiliations:** a Department of Molecular Physiology and Biological Physics, School of Medicine, University of Virginia, Charlottesville, VA 22903, USA; b Center for Membrane and Cell Physiology, School of Medicine, University of Virginia, Charlottesville, VA 22903, USA; c Translational Neurodegeneration Section „Albrecht Kossel“, Department of Neurology, University Medical Centre Rostock, University of Rostock, Rostock, Germany; d Dresden High Magnetic Field Laboratory (HLD-EMFL), Helmholtz-Zentrum Dresden-Rossendorf (HZDR), D-01328 Dresden, Germany; e University of Würzburg, Theodor Boveri Institute, Department of Bioinformatics II, Am Hubland, 97074 Würzburg, Germany; f Department of Biology, Graduate School of Arts and Sciences, University of Virginia, Charlottesville, VA 22902, USA; g College of Dental Medicine, Columbia University, New York, NY 10032, USA; h Department of Biomedical Engineering, School of Medicine, University of Virginia, Charlottesville, VA 22902, USA; i Goethe University Frankfurt, Institute for Molecular Biosciences, Max-von-Laue-Str. 13, 60438 Frankfurt am Main, Germany; j Department of Cell Biology, School of Medicine, University of Virginia, Charlottesville, VA 22903, USA; k German Center for Neurodegenerative Diseases (DZNE) Rostock/Greifswald, 18147 Rostock, Germany; l Center for Transdisciplinary Neurosciences Rostock (CTNR), University Medical Center Rostock, Rostock, Germany

**Keywords:** Amyotrophic lateral sclerosis, Induced pluripotent stem cells, Axon degeneration, RNA sequencing, Axonal transcriptome, DNA damage, PLK1, Cell cycle, ECM, Axonal outgrowth

## Abstract

Mutations in *FUSED IN SARCOMA (FUS*) cause juvenile-onset amyotrophic lateral sclerosis (ALS). Early pathogenesis of *FUS*-ALS involves impaired transcription and splicing, DNA damage response, and axonal degeneration. However, the molecular pathophysiology and the link between somatic and axonal phenotypes are still poorly understood. We evaluated whether compartment-specific transcriptome differences could distinguish and drive early axonal degeneration. We used iPSC-derived motor neurons (MNs) coupled with microfluidic approaches to generate RNA-sequencing profiles from axonal and somatodendritic compartments. We demonstrate that the axonal transcriptome is unique and distinct, with RNA metabolism, extracellular secretion, and matrix disassembly pathways particularly enriched in distal axonal compartments.

*FUS* mutation leads to changes in distinct pathways that were clustered in only a few distinct protein-protein interaction (PPI) networks. Somatodendritic changes upon *FUS* mutation include WNT signaling, mitochondrial, extracellular matrix (ECM)-, and synapse-related functions. In contrast, analysis of the axonal transcriptome in mutant MNs centers on the *PLK1* pathway, mitochondrial gene expression, and regulation of inflammation. Comparison to CLIP-seq data revealed a significant enrichment for *PLK1* and DNA replication pathways in axons. *PLK1* upregulation did not activate cell-cycle re-entry but contributed to mutant MNs survival, and its inhibition increased neuronal cell death. We propose that upregulation of *PLK1* represents an early event in the pathogenesis of ALS and could act in response to DNA damage, mitochondrial damage, and immune response activation in the affected cells. Additionally, downregulation of ECM pathways in the somatodendritic compartment and axons could explain strongly compromised dynamics of axonal outgrowth. Overall, we provide a novel valuable resource of the potential targets and affected processes changed in the specific compartments of *FUS-*ALS motor neurons.

## 1. Introduction

Amyotrophic lateral sclerosis (ALS) is a severe and incurable disease that is characterized by degeneration of upper and lower spinal motor neurons (MNs) usually starting by denervation at the distal axons (Taylor et al., 2016). The identification of a number of genetic factors associated with ALS and the ability to model this disease in various cell cultures and in vivo model systems have contributed enormously to our understanding of pathogenic mechanisms underlying the disease (Hawrot et al., 2020; Wang et al., 2023). While ~90% of ALS patients suffer from apparently sporadic disease, ~10% show classical Mendelian heritability with mutations found in over 40 genes (Wang et al., 2023). Mutation of the *FUSED IN SARCOMA (FUS)* gene belong to one of the most common forms of familial ALS and are particularly prevalent in young patients (Kwiatkowski et al., 2009).

*FUS* encodes a primarily nuclear protein that contains RNA binding- and low-complexity-domains (LCD) and has been implicated in regulating transcription, DNA damage, RNA splicing, nucleocytoplasmic and axonal trafficking, phase-separation dynamics of ribonucleoprotein assemblies, and metabolism (Ling et al., 2013). Many of the disease causing mutations are located within nuclear localization sequence (NLS) (Naumann et al., 2019), and cytoplasmic mislocalization of *FUS* is a pathophysiological hallmark of *FUS*-ALS (Dormann and Haass, 2013). *FUS* binding was mapped to multiple sites on chromosomes and more than a thousand long coding mRNAs, where it was shown to regulate their transcription and splicing (Ishigaki et al., 2012; Lagier-Tourenne et al., 2012; Reber et al., 2016; Rogelj et al., 2012; Yang et al., 2014). This ability of *FUS* to regulate many identified binding targets is likely to be central to ALS progression and development. However, the detailed contribution of such potentially global misregulation, as well as hierarchy of contributing pathways in disease progression remains unclear. Some of the downstream defects in ALS clearly converge on or are triggered by the formation of toxic proteins or ribonucleoprotein (RNP) aggregates (Brauer et al., 2018). In this respect ALS is similar to other neurodegenerative diseases, where multiple cellular mechanisms drive the vulnerability of their specific subsets of neuronal cells (Brauer et al., 2018).

Previous studies have shown that changes in mRNA and protein levels can occur in specific neuronal compartments, such as distal axons, and thus these changes could provide insight into the underlying mechanisms of ALS (Holt and Schuman, 2013; Perry and Fainzilber, 2014). Interestingly, the pathogenesis of ALS is often referred to as axonopathy, with particular vulnerability of distal axons/neuromuscular junctions and muscle denervation being the first symptoms of the disease (Clayton et al., 2024). It has been shown that this is particularly true for *FUS*-ALS (Guo et al., 2017; Naumann et al., 2018; Wongworawat et al., 2020). Induced pluripotent stem cell (iPSCs)-derived MNs of *FUS-*ALS exhibited distal axonopathy with depolarized distal axonal mitochondria prior to a dying-back and nerve cell loss. *FUS*-ALS MNs also accumulated DNA damage, and DNA damage induction was sufficient to induce distal axonal mitochondrial depolarization (Naumann et al., 2018; Wang et al., 2018). While axonal ATP levels were lower in general in iPSC-derived MNs, no axonal compartment specific differences in ATP levels in MNs with *FUS*-ALS mutations have been yet detected (Zimyanin et al., 2023), nor have reductions in ATP consumption been observed (Kodavati et al., 2024; Vandoorne et al., 2019). Thus, the exact mechanisms by which nuclear DNA damage affects axonal mitochondrial integrity are so far not understood.

Recent advances in high-throughput transcriptomic profiling based on next-generation RNA-sequencing (RNA-seq) approaches have provided insights into expression patterns and mechanisms of ALS pathogenesis in healthy and diseased conditions (Dash et al., 2022, 2024; Masuda et al., 2016; Ziff et al., 2023). However, the transcriptome profiles often show surprisingly little overlap in what should be comparable or at least converging disease models, partially due to potential genetic background differences and purity of cell populations (Dash et al., 2020). These global quantifications also lack information about local changes of the transcriptome in different cellular compartments. Only few reports exist of RNA-seq methods used to characterize axonal transcriptome: This was shown in studies on primary mouse MNs and primary mouse dorsal root ganglia (DRG) (Briese et al., 2016; Di Paolo et al., 2021; Farias et al., 2020; Minis et al., 2014; Rotem et al., 2017; Suzuki et al., 2020) and mouse embryonic stem cell (mESC)-derived motor neurons (Nijssen et al., 2018). To the best of our knowledge, only two studies have so far investigated iPSC-derived MNs (Garone et al., 2023; Nijssen et al., 2018). Nijssen and colleagues investigated somatic vs. axonal compartments using microfluidic chambers in healthy and *SOD1* mutant mESC-derived MNs. While Garone et al. studied somatic vs. neuritic compartments of iPSC-derived MNs of FUS^wt^ and FUS^P525L^ using modified Boyden chambers (Garone et al., 2023; Nijssen et al., 2018). Only the former of these studies distinguished between dendrites and axons in their transcriptome.

The aim of the study thus was to comparatively investigate transcripts and molecular pathways critical for axonal function and degeneration in (*FUS*-)ALS by analyzing somatodendritic vs. axonal transcriptome of FUS^wt^ and FUS^mut^ iPSC-derived MNs. Using isogenic CRISPR/Cas9 edited iPSC-lines harboring *FUS*^*P525L*^ mutation (Japtok et al., 2015; Naumann et al., 2018), coupled with microfluidic approaches we profiled and compared transcriptomes in somatodendritic (SD) and axonal compartments. Our differential analysis of mRNA expression in SD and axon compartments highlighted a number of critical pathways including DNA damage, mRNA splicing and mitochondrial functions. Furthermore, we showed an upregulation of the cell cycle related gene polo-like kinase 1 (*PLK1*) in mutant MNs and this activation has important implications on survival of *FUS*-ALS MNs. We propose that upregulation of these genes could function at the interplay connecting DNA damage activation in the soma to cytoskeleton and mitochondrial defects, as well as innate immune response activation in distal axons.

## 2. Methods

### 2.1. Cell lines

For RNA-seq we used the two pairs of isogenic cell lines FUS^WT^-eGFP^het^ and FUS^P525L^-eGFP^het^ that were generated, described and characterized in Naumann, Pal et al., 2018 and Marrone et al., 2018 (Marrone et al., 2018; Naumann et al., 2018). MNs were generated following the protocol established and used in these papers and both are heterozygous for the tagged gene.

All other cell lines were also from our previous studies and were obtained from skin biopsies of patients or healthy volunteers or generated using CRISPR/Cas9 mutagenesis and have been described before (Supplementary Table 10) (Japtok et al., 2015; Naumann et al., 2018; Peter et al., 2017; Petters et al., 2020). The performed procedures were in accordance with the Declaration of Helsinki (WMA, 1964) and approved by the Ethical Committee of the Technische Universität Dresden, Germany (EK 393122012 and EK 45022009) and by the Swedish Ethical Committee for Medical Research (Nr 94–135 to perform genetic research, including for *FUS*, and Nr 2018–494–32 M to perform in vitro lab studies on cell lines derived from patients with ALS). Written informed consent was obtained from all participants, including for publication of any research results. All fibroblast lines were checked for Mycoplasma spp. before and after reprogramming, and afterward, routine checks for Mycoplasma were conducted every three to six months. We used the Mycoplasma detection kit according to the manufacturer’s instructions (Firma Venor GeM, No 11–1025).

### 2.2. Differentiation of NPCs to MNs

The generation of human NPCs and MNs was accomplished following the modifications protocol from Reinhardt et al. (2013) and described also in Naumann, Pal et al., 2018 (Naumann et al., 2018; Reinhardt et al., 2013). In brief, iPSCs colonies were collected and stem cell medium, containing 10μM SB-431542, 1 μM Dorsomorphin, 3 μM CHIR 99021 and 0.5 μM SAG (Cayman; 11,914) was added. After 2 days hESC medium was replaced with N2B27 consisting of the aforementioned factors and DMEM-F12/Neurobasal 50:50 with 1:200 N2 Supplement, 1:100 B27 lacking Vitamin A and 1% penicillin/streptomycin/glutamine. On day 4150μM ascorbic acid was added while Dorsomorphin and SB-431542 were withdrawn. 2 days later the EBs were mechanically separated and replated on Matrigel coated dishes. For this purpose, Matrigel was diluted (1:100) in DMEM-F12 and kept on the dishes over night at room temperature. Possessing a ventralized and caudalized character the arising so called small molecule NPCs (smNPC) formed homogenous colonies during the course of further cultivation. It was necessary to split them at a ratio of 1:10–1:20 once a week using Accutase for 10min at 37°C and were not used beyond 10 consecutive passages. *FUS* iPSC and control cell lines were recently karyotyped using the HumanCytoSNP-12v array. All clones showing pathological SNPs were excluded.

Final MN differentiation was induced by treatment with differentiation medium with 0.5 μM SAG, 0.2 mM Ascorbic Acid, 1 μM retinoic acid (RA), 1 ng/ml BDNF, 1 ng/ml GDNF 0.5μM SAG in N2B27 and changed every 2 days. After 6 days differentiation media was changed to maturation media containing 5 ng/ml Activin A, dbcAMP 0.1 mM, 2 ng/ml BDNF, 2 ng/ml GDNF, 1 ng/ml TGFß-3, 0.2 mM Ascorbic Acid. On day 7–10 another split step was performed to seed them on a desired cell culture system and kept in the maturation media without Activin A but with one-time addition of 2.5 μM DAPT. Following this protocol, it was possible to keep the cells in culture for over 2 months.

### 2.3. Growth of MNs in microfluidic chambers

The MFCs were purchased from Xona (RD900 with 900 μm long microgrooves). At first, Nunc glass bottom dishes with an inner diameter of 27 mm were coated with Poly-L-Ornithine (Sigma-Aldrich P4957, 0.01% stock diluted 1:3 in PBS) overnight at 37 °C. After 3 steps of washing with sterile water, they were kept under the sterile hood for air drying. MFCs were sterilized with 70% Ethanol and also left drying. Next, the MFCs were dropped onto the dishes and carefully pressed on the glass surface for firm adherence. The system was then perfused with Laminin (Roche 11,243,217,001, 0.5 mg/ml stock diluted 1:50 in PBS) for 3 h at 37 °C. For seeding cells, the system was once washed with medium and then 10 μl containing a high concentration 3 × 10^5^ cells (3 × 10^7^ cells/ml) were directly injected into the main channel connecting two wells. After allowing for cell attachment over 30–60 min in the incubator, the still empty wells were filled up with maturation medium. This method had the advantage of increasing the density of neurons in direct juxta-position to microchannel entries whereas the wells remained cell-free, thereby reducing the medium turnover to a minimum. To avoid drying out, PBS was added around the MFCs. Two days after seeding, the medium was replaced in a manner which gave the neurons a guidance cue for growing through the microchannels. Specifically, a growth factor gradient was established by adding 100 μl N2B27 with 500 μM dbcAMP to the proximal seeding site and 200 μl N2B27 with 500 μM dbcAMP, 10 ng/μl BNDF, 10 ng/μl GDNF and 100 ng/μl NGF to the distal exit site. The medium was replaced in this manner every third day. After 7 days, the first axons began spreading out at the exit site and cells were typically maintained for up to six weeks. MFCs with varying length of the channels and percentage of crossed channels (out of 121) was calculated for the axonal outgrowth experiment.

### 2.4. Axonal growth analysis in microfluidic chambers (MCFs)

**Fig. 1.**
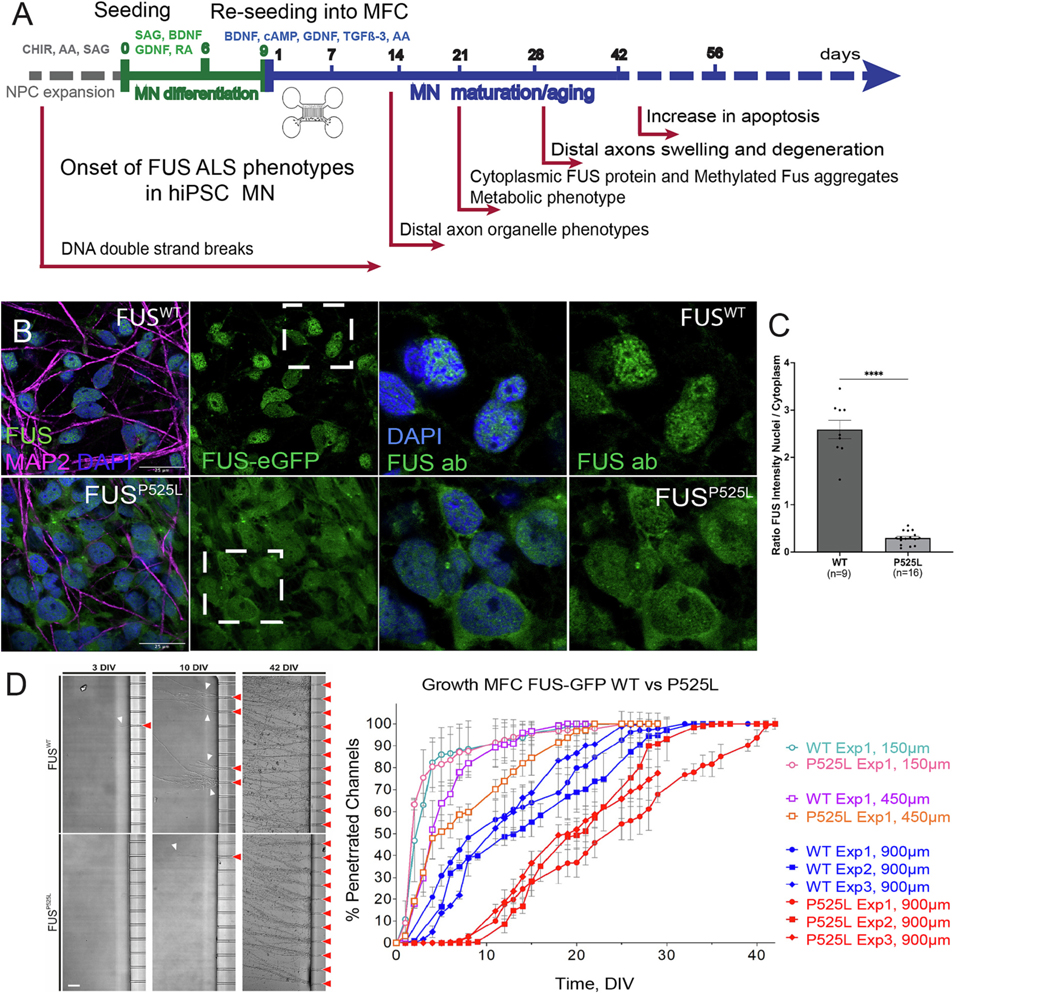
Compartmentalized transcriptomics of FUS^P525L^ MNs. (A) Timeline and schematic outline of the MN differentiation protocol starting from neural precursor cells and undergoing sequential differentiation and maturation. Time points for seeding into MFC and onset of previously reported *FUS*-ALS-related phenotypes are indicated under the timeline. (B-C) Mature cultured MNs showing mis-localization of mutant FUS^P525L^ protein from the nucleus to the cytoplasm, as seen by fluorescence imaging of *FUS*-eGFP tagged isoforms (left) or by immunostaining with FUS antibody (right) and quantified in (C). Field of view images analyzed *n*^wt^ = 9, *n*^FUSP525L^ = 16 from 3 independent experiments. *p*-value *<* 0.0001 with unpaired parametric Studenťs *t*-test. (D) Representative images showing axonal crossing to the distal compartment of the microfluidic device at DIV3, DIV10 and DIV42 and quantification of this analysis. Quantification of axonal growth through the MFCs with varying length of channels estimating a percentage of crossed channels with time. *n* = 3 technical replicas per individual experiment for each curve. The standard deviation across the three replica MFCs is displayed as error bars, the experiment was performed three times for RD900 MFCs. Error bars are SEM in (C) and standard deviation in (D). Significance assessed with unequal variance student *t*-test. * *p* ≤ 0.05, ** *p* ≤ 0.01, *** *p* ≤ 0.001, **** *p* ≤ 0.0001.

Spinal iPSC-derived FUS^WT^ and FUS^P525L^ motor neurons (MNs) were differentiated and seeded at 0 days in vitro (0 DIV) into the proximal compartment of Xona microfluidic chambers (MFCs) as described above and shown in Fig. 1D. Seeding was performed into three replica MFCs per cell line either of 150 μm (RD150), 450 μm (RD450), or 900 μm (RD900) channel length. To analyze the growth of axons protruding from the proximal channel entries to the distal exits, we chose a label-free method to avoid any growth perturbation due to chronic exposure of the cells to dye molecules for the daily growth assessment (with some gaps over weekends) over the time course from 0 to 42 DIV (Fig. 5A). Furthermore, we favoured a swift method for all replica MFCs as removal of the cell samples from the incubator for the daily growth assessment for too long will perturb the axon growth due to the lengthy interruptions in temperature and CO2 maintenance. Therefore, to minimize the time requirement, we refrained from acquiring images but swiftly inspected all 121 channel exits of each MFCs by eye under a basic cell culture microscope at 10× magnification with brightfield transmission light. If at least one axon was reaching out from the exit into the open distal compartment, the respective channel was counted as “penetrated” using a manual tally clicker. The obtained total count was used to calculate the percentage of penetrated channels at each time point as measure of axon growth. The standard deviation across the three replica MFCs is displayed as error bars, the experiment was performed three times for RD900 MFCs (Fig. 5A).

### 2.5. RNA extraction and sequencing analysis

For transcriptome sequencing (RNA-seq), MNs of the cell lines FUS^WT^ and FUS^P525L^ were generated in biological triplicates (3 independent differentiations side by side) and two-week-old microfluidic chambers (MFC) with abundant outgrowth of axons were selected for RNA isolation. A total of 7–10 MFC were used per each cell line condition to extract the total RNA. Briefly, both SD and axonal compartments were washed with PBS and were lysed by direct application of Qiazol lysis buffer (Qiagen, Hilden, Germany) from the kit into chambers. Following that, RNA purification was performed using an RNeasy Mini Kit (Qiagen, Hilden, Germany) according to the manufacturer’s instructions including a column DNase digest for all samples. RNA quantity and quality was evaluated by Agilent 2100 Bioanalyzer using Agilent RNA 6000 Pico Kit (Agilent Technologies, CA, USA). For RNA quality assessment, fragment size distribution (DV200; percentage of RNA fragments with a length *>* 200 nucleotides) was calculated for each sample using 2100 Expert software. Samples with DV200 ≥ 60% were subjected to RNA-seq. mRNA was purified from 10 ng total RNA and exponentially amplified using Takara Bio SMARTer-Seq (Clontech/Takara Bio USA) according to the manufacturer’s instructions. RNA libraries for both SDs and axon RNA-seq were prepared using a SMARTer HV chemistry, Ultra mRNA-Seq Library Prep Kit-v3 (Clontech/Takara Bio USA) following manufacturer’s recommendations and processed on an Illumina HiSeq 2500 system (polyA[+] RNA-seq), resulting in 75 bp single-end sequencing mode to an average depth of 30 million fragments per library. Sequencing libraries were constructed and sequenced at next-generation sequencing facility (Dresden-concept Genome Center), on Illumina HiSeq2500 platform.

FastQC (v0.11.3) measure was used to perform a basic quality control (QC) on the sequence data. Reads were mapped to the human reference genome hg38/GRCh38 (obtained from EnsEMBL v81) using the aligner GSNAP (v2017–11-15) (with splice-junction support from annotated genes (EnsEMBL v81)) (Wu and Nacu, 2010). Further quality control on mapped reads, rRNA content, coverage of exonic/intronic/intergenic regions and number of detectable genes was done with RNA-SeQC (v1.1.8) (DeLuca et al., 2012). A table of raw read counts per gene was obtained based on the overlap of the uniquely mapped reads with annotated human genes (EnsEMBL v81) using featureCounts (v1.5.2) (Liao et al., 2014). Normalization of the raw read counts based on library size was performed with the DESeq2 R package (v1.16.1) (Love et al., 2014). Principal component analysis (PCA), sample-to-sample Euclidean distance as well as Pearson’s and Spearman’s correlation coefficients were computed based on the normalized gene expression level using R. For testing for differential expression with DESeq2, the count data was fitted to the negative binomial distribution and adjusted *p*-values for the statistical significance of the fold change (FC) were adjusted for multiple testing with the Benjamini-Hochberg correction for controlling the false discovery rate (FDR) accepting a maximum of 5% false discoveries (adjusted *p*-value ≤0.05) and log2fold change (FC) ≥ 1. Heatmap and unsupervised hierarchical clustering of DEGs in axon and soma samples of control/mutant MNs were generated using R package. Heatmap and unsupervised hierarchical clustering of DEGs in axon and soma samples of control/mutant MNs were generated using R and *Partek*™ *Flow*™ software, v11.0. Data are deposited at NCBI/GEO under the accession number GSE276214.

### 2.6. Use of published datasets

All data used in cross-comparisons of datasets were obtained from the NCBI/GEO database. The GEO accession numbers and their respective study references for the axonal transcriptomic studies were as follows: GEO: GSE66230 (Briese et al., 2016), GEO: GSE121069 (Nijssen et al., 2018) (Supplementary Table S5). In all cases for RNA-seq, FASTQ data was processed and DESeq2 normalization values were calculated using the pipeline described above in *Partek*™ *Flow*™ software, v11.0, to avoid differential mapping bias across sample datasets.

### 2.7. Functional annotation of DEGs by GO and KEGG analysis

To better interpret the possible pathways and target genes of the relevant profiles of the SD and axon-enriched genes analyzed in *FUS-*ALS, GO functions and pathway (Reactome and Kyoto Encyclopedia of Genes and Genomes (KEGG)) enrichment analyses of candidate genes were carried out by utilizing the Metascape and EnrichR, integrated online tools for gene list annotation and biological analysis (Xie et al., 2021; Zhou et al., 2019). Metascape integrates several functional databases, such as GO, KEGG and Uniprot to analyze multiple gene sets simultaneously. The DEGs were analyzed and visualized by Metascape with the criteria of minimum overlap ≥3, *p*-value ≤0.05, and minimum enrichment score *>* 1.5. We utilized EnrichR (enrichment testing across all GO functions, pathways) with Fisher’s exact test to conduct the functional enrichment analysis with the DEGs. In Metascape and EnrichR analysis, we combined the signaling pathways from two libraries, including KEGG and Reactome, to create a single analysis route. Only the important paths for which the *p*-values ≤0.05 were evaluated and considered after deleting duplicate pathways. For functional GO annotations, we looked at the GO biological process (BP), GO molecular function (MF), and GO cellular component (CC) datasets in Metascape and EnrichR and selected the most important GO terms based on set criteria and with a *p*-value ≤0.05. Gene set enrichment analysis (GSEA) (Subramanian et al., 2005) was performed on the normalized gene counts (DESeq2) of RNA-seq data (mutant and control) using the GSEA function in the *Partek*™ *Flow*™ software, v11.0. The used gene sets for testing were the GO-term derived gene set database (BP, MF, and CC) and KEGG pathway database, and the significant gene sets were defined as those with a normalized enrichment score (NES) *>* 1 and *p* ≤ 0.05.

Venn diagrams of target genes showing axon-soma differential expression (FC ≥ 1 or FC ≤ −1, adjusted *p*-value ≤ 0.05) in ALS model cultures were selected as the candidate target genes for further downstream analysis and extracted via the online tool InteractiVenn.

### 2.8. PPI network construction and identification of key genes

The protein-protein interaction (PPI) networks were generated with the Search Tool for the Retrieval of Interacting Genes (STRING) database v12.0 as well as stringApp of Cytoscape v3.10.1 software (Shannon et al., 2003; Szklarczyk et al., 2015). In the present study, PPI network of axon (137) and SD (166) differentially expressed genes (DEGs) was generated with a medium confidence score of 0.4 (confidence score ≥ 0.4), disconnected nodes were excluded from the network. To further strengthen the PPI core network of identified SD upregulated and axon downregulated DEGs, 5–10 direct binding partners were added to reveal functional interactions between the deregulated proteins. Functional enrichment analyses were carried out using STRING database. The nodes indicate the DEGs and the edges indicate the interaction (databases, high-throughput experiments, co-expression/co-occurrence, text mining, neighbourhood genes) between two proteins. The PPI network was subsequently imported into Cytoscape for further visualization and analysis. Furthermore, the rank and score of these key genes/clusters were identified based on a network topological property using NetworkAnalyzer v4.4.8 plugin of the Cytoscape. The statistical enrichment analyses in STRING indicated that the DEGs were significantly enriched in PPI networks (*p*-value ≤0.05).

### 2.9. Splicing analysis

Splicing analysis was performed using MAJIQ (version 2.2) (Vaquero-Garcia et al., 2023). We used the majiq-build function to construct a single splice graph over all replicates and conditions (FUS^WT^ and FUS^P525L^). Majiq-deltapsi was used to quantify local splicing variations (LSVs) between X and Y and Z datasets. Identified LSVs from both comparisons were considered significantly regulated with a difference in junction usage (percent selected index, PSI) of DPSI *>*5% and a changing probability *>*50%. Non-regulated LSVs were considered with DPSI *<*2% and an LSV changing probability of 0%. Both regulated and nonregulated LSVs were subsequently stratified to the binary level and were classified into the events ‘intron-retention’, ‘alternative 3’ splice site’, ‘alternative 5’ splice site’ and ‘cassette exon’. This was done by combining source and target LSV junctions for a given event, requiring both LSVs to be regulated as defined above. Cassette exon events were further sub-classified into ‘simple cassette exon’, ‘alternative first exon’, ‘alternative last exon’ and ‘complex events’ using the same methodology.

### 2.10. CLIPseq analysis

All data used in *FUS* CLIP analysis were obtained from Kügelgen et al., 2025 (von Kügelgen et al., 2025). Briefly, *FUS* CLIP targets were extracted (*>*1 CLIP binding site) and gene lists were then used to construct a Venn diagram to visually compare and highlight common genes across axonal and *FUS* CLIP datasets. To determine the statistical significance of the overlap, we used the total number of genes (background set, *N* = 35,039) that were actually detected in our RNA-seq experiment (FUS^P525L^ versus FUS^WT^) and calculated the expected number of overlapping genes using axonal DEGs and *FUS* CLIP sequencing targets. *p*-value (*p* ≤ 0.05) was calculated using Fisher’s exact test to indicate the statistical probability. Fold enrichment (observed overlap (29)/expected overlap (17.21)) over the expected background of 35,039 expressed genes was calculated to demonstrate the magnitude of overlap. To further interpret the biological meaning of this significant overlap, we performed pathway and PPI enrichment analysis on these 29 specific genes using EnrichR and STRING databases (*p* ≤ 0.05).

### 2.11. Cell cycle analysis

Cell cycle analysis for cultured NPCs was performed after 24 h of incubation with DMSO or BI2536 (a potent and selective *PLK1* inhibitor, Selleckchem) treatment with concentrations ranging from 3 nM–1000 nM. Briefly, the cells (~1–2 × 10^6^ cells/ml) were collected in 500 μl and fixed by the dropwise addition of 500 μl of ice cold 70% ethanol (−20 °C) whilst gently mixing and stored at 4 °C overnight. The next day, the ethanol-PBS 1:1 solution was removed by pelleting the cells (300 ×g, 5 min, 4 °C), followed by two washing steps with cold PBS. The cells were resuspended in PBS containing 0.5 ml/tube FxCycle™ PI/RNase staining solution (Propidium iodide/RNase, Thermo Fisher Scientific) following the manufacturer’s guidelines and incubated in the dark at 37 °C for 30 min. For each sample 50,000 events were acquired and determination of NPCs distribution among G0/G1, S, G2/M cell cycle phases of each group was performed at 488 nm using a FACSCalibur flow cytometer (BD Biosciences, San Jose, CA, USA). Data were analyzed by BD FlowJo software v10.7.2 (BD Pharmingen, San Diego, CA, USA) and the gating strategy is shown in Supplementary Fig. S7.

For cultured MNs (DIV21), the cells (~1–2 × 10^6^ cells/sample) were harvested (Accutase (Sigma) for 5 min at 37 °C) and washed twice with PBS. All procedures were performed using FACS buffer (PBS, 1 mM EDTA, 5% normal goat serum, 10% FBS). After addition of FACS buffer, the cell suspension was centrifuged (300 ×*g*, 5 min, 4 °C), washed twice with cold PBS, and fixed in ice-cold 70% ethanol. Intracellular staining was done after 2 h of fixation and permeabilization with ethanol at 4 °C using the polyclonal chicken anti-MAP2 primary antibody (1:2000, Abcam) and incubated (in 100 μl FACS buffer) for 60 min on ice. The cells were washed with FACS buffer and labeled with a secondary Alexa Fluor 647-conjugated (infrared) antibody (goat anti-chicken IgY, 1:1000, Invitrogen) and incubated again for 45 min on ice. In order to exclude non-specific binding of the Alexa Fluor-labeled secondary antibody, control cells were additionally stained with the secondary antibody without using the primary antibody. For MAP2 quantification, the number of cells that were positive for the secondary antibody was subtracted from the MAP2+ secondary antibody stained cells. Cells were washed and resuspended in 500 μl FACS staining/buffer solution consisting of 500 μl/sample FxCycle™ PI/RNase staining solution (Thermo Fisher Scientific) according to the manufacturer’s instructions and incubated in the dark at 37 °C for 30 min. Finally, flow cytometry measurements were taken at excitation wavelength of 488 nm using a FACSCalibur flow cytometer (BD Biosciences, San Jose, CA, USA). For each sample 50,000 events were acquired and data analysis to detect the MAP2-positive MNs cell cycle (G0/G1, S, G2/M) distribution of each group was performed using BD FlowJo v10.7.2 software (BD Pharmingen, San Diego, CA, USA). Specific subpopulations as well as related flow cytometry gating scheme were defined as outlined in the Supplementary Figs. S8, S9.

### 2.12. Apoptosis/necrosis analysis

Apoptotic/necrotic cells were quantified by flow cytometry using an Annexin V/PI and NeuO multicolor staining procedure (BioLegend and STEMCELL Technologies) according to the manufacturer’s instructions. In brief, DMSO or BI2536 (100 nM, Selleckchem, 72 h, 37 °C) treated MNs (DIV21) were labeled with NeuroFluor™ NeuO dye (STEMCELL Technologies) with Ex/Em of 468/557 nm that selectively labels neurons in live culture. The dye was diluted in the respective culture medium to the concentration of 0.1 μM and the medium was applied directly to the cells, which were then incubated for 1 h at 37 °C. Next, the labeling medium was removed and replaced with fresh culture medium and the cells were incubated for another 90 min. After that, the cells were washed once with FACS buffer (PBS, 1 mM EDTA, 5% normal goat serum, 10% FBS) and detached with Accutase (Sigma, 5 min at 37 °C). The detached cells (from culture medium and Accutase treatment) were then washed twice with cold FACS buffer (300 ×g, 5 min, 4 °C) and the cell pellet was resuspended at the density of 1 × 10^6^ cells/ml in 100 μl Annexin V binding buffer (BioLegend, #422201). All the following steps were performed on ice. Cell suspension was then filtered through the cell strained cap with 35-μm nylon mesh. Subsequently, 5 μl APC Annexin V (BioLegend, #640919) was added to cell suspension and the cells were incubated for 15 min in the dark (RT), followed by the addition of 10 μl of 0.5 mg/ml PI (BioLegend, #421301) to identify dead cells and incubation of the cells for 5 min at 4 °C. After incubation period, 400 μl Annexin V binding buffer was added and APC Annexin V fluorescence and PI fluorescence were then detected using a Cytek™ Aurora spectral flow cytometer running on the SpectroFlo software v2.2.0.3 (Cytek Biosciences, Fremont, CA, USA). At least 50,000 events were recorded for each sample and the acquired apoptosis/necrosis data were analyzed using FlowJo v10.7.2 software (BD Pharmingen, San Diego, CA, USA). Gating strategies are shown in Supplementary Figs. S10, S11. Unstained and single stained cells were used as controls and measured in every single experiment. Annexin V^−^ /PI^−^ cell populations were indicated living cells, Annexin V^+^/PI^−^ were considered early apoptotic cells, whereas those that were positive for both Annexin V^+^/PI^+^ were identified as necrotic or late apoptotic cells. The apoptotic rates were determined as the percentage of the total cell population. All FACS measurements including cell cycle analysis were carried out in the Core Facility for Cell Sorting and Cell Analysis, University Medical Centre Rostock, Germany.

### 2.13. PCR PLK1 mRNA

mRNA was prepared from a DIV21 cultures of control and P525L MN cultures grown in microfluidic chambers using Qiagen mRNA mini kit. cDNA was generated using Superscript RT DNA polymerase (Thermo Fischer Scientific). We used IQ SYBR Green Supermix (Biorad) and mRNA preps from 3 separate mRNA samples were run in triplicates, and were normalized against two different human house-keeping genes (*RBS18* and *HPRT*). Following primers were used: *PLK1*: GCACAGTGTCAATGCCTCCAAG and GCCGTACTTGTCCGAATAGTCC, *RBS18*: GCAGAATCCACGCCAGTACAAG and GCTTGTTGTCCAGACCATTGGC and *HPRT*: CATTATGCTGAGGATTTGGAAAGG and CTTGAGCACACAGAGGGCTACA.

### 2.14. Western blot

Protein samples for Western blot were prepared from MN cultures grown in multi well plates and lysed using Mild protein lysis buffer and sonication. Standard mild lysis buffer is 50 mm Tris-HCl, pH 7.8, 150 mm NaCl, 1% Triton X-100, 10% glycerol, 25 mm NaF, 1× Roche cOmplete™ Mini tablets protease inhibitor (5–10 μM is usually sufficient). Samples were prepared from independent experiments. Purified anti-PLK1 Antibody (Mouse) (1:1000) (#627701, Biolegend) and PLK1 (208G4) Rabbit mAb #4513 (Cell Signaling technology, 4513S) (1:500). Either Mouse a-Tubulin antibody or GAPDH (14C10) Rabbit mAb (HRP Conjugate) #3683 Cell Signaling was used as a loading controls. Secondary antibodies were either IRDye® 800CW Goat anti-Rabbit IgG Secondary Antibody and IRDye®, 680RD Goat anti-Mouse IgG Secondary Antibody (LicorBio) or HRP-conjugated anti-mouse and anti-rabbit with ECL detection reagents.

### 2.15. Cell culture and treatments

Etoposide treatment of HEK cells and MNs: HEK cells were grown in DMEM/F12, 10% FBS, Pen/Strep media with Non-essential amino acids (from 100× stock) and Sodium Pyruvate (100× stock). Cells were treated with 5 μM, 25 μM or 50 μM Etoposide and either lysed in RIPA buffer with sonication or fixed in 4% PFA for immunohistochemistry. MN cultures were grown for 14dpf and treated overnight with DMSO as control or 25 μM Etoposide. Cells were then fixed in 4% PFA and immune stained. Mean intensity of MNs per cell was counted within an ellipsoid ROI covering most of the cell and were averaged per image and condition. *n* = 3 technical replicates per condition, with minimum 3 field of view images per replica and 7–10 cells per image. For HEK cells, Mean intensity per cell was counted within an ellipsoid ROI covering most of the cells. n = 3, with *>*40 cells measured per condition across 5–7 images.

*PLK1* inhibitor treatment: neurons were differentiated from iPCS-derived NPCs as described above and were treated with 1 μM BI2536 for 24 h, 2 μM Etoposide for 1 h, or with the equivalent volume of DMSO. Neurons were then fixed in 4% paraformaldehyde for 20 min at 37 °C. Afterwards, neurons were permeabilized in 0.2% Triton X-100 for 10 min. Then, MNs were washed in tris-buffered saline containing 1% Tween 20 for 10 min for 3 times and subsequently blocked in Pierce Protein-free blocking buffer (ThermoFisher: 37572) for 1 h at room temperature.

### 2.16. Immunostainings

PLK1 staining: MNs were grown in multi well cell plates for 14 dpm. Imunnostaining for PLK1 was done with polyclonal PLK1 (208G4) Rabbit mAb #4513 (Cell Signaling technology, 4513S). All cell nuclei were counted by ‘analayze particles’ plugin in FIJI with manual correction. PLK1 high signal cells were counted manually. High PLK1 signal was considered an intensity which had at least double of mean intensity value averaged across 25 random cells. Mean intensity per cell was counted within an ellipsoid ROI covering most of the cell. At DIV14: *n* = 3 experiments. Total cell numbers and high PLK1 positive cells within 3–10 field of view images analyzed per condition, per experiment.

DNA damage foci: The primary antibodies α-MAP2 (Abcam: ab5392; 1:100,000) and α-γH2AX (Millipore: 05–636; 1:500) were diluted in Pierce Protein-free blocking buffer (ThermoFisher Scientific: 37572) and incubated over-night at 4 °C on a shaker. Samples were then rinsed in TBS for 10 min three times and the secondary antibodies Alexa Fluor488 α-chicken (Invitrogen: A11039) and Alexa Fluor-568 anti-rabbit (Invitrogen: A11036) were diluted 1:5000 in TBS and incubated for 1 h at room temperature, on a shaker. Samples were rinsed three times in TBS for 10 min and then mounted in DAPI Fluoromount-G mounting medium (Southern Biotechnology: 0100–20).

*FUS* nuclei localisation was done with primary α-FUS antibody (Sigma Atlas, AMAB90549–100UL, 1:5000). We used FIJI to create nuclei object masks using DAPI channel. We then measured ratio of fluorescent intensity of *FUS* signal within the DAPI mask and intensity of *FUS* in the whole image after subtracting the intensity of *FUS* in the nucleus.

### 2.17. Microscopy

Images were acquired on a Zeiss inverted AxioObserver.Z1 microscope with LSM 900 module and high-resolution Airyscan 2 module, using a 63 × 1.4 NA plan apochromat objective. Data acquired from microscopy were evaluated using Fiji v1.53t.

### 2.18. Statistical analysis

All statistical analyses including Hierarchical clustering (with Euclidean distance measure and average linkage clustering), QC assessment, and cross-comparison studies were performed using R-studio software and *Partek*^*™*^ *Flow*^*™*^ software, v11.0. Differential expression was analyzed by applying a adjusted *p* ≤ 0.05 to the DESeq2 results followed by false discovery rate (FDR) step-up for multiple comparisons. Genes with FDR/adjusted *p*-value ≤ 0.05 and log2FC ≥ 1 or log2FC ≤ −1 were considered as significant thresholds for the identification of DEGs. All of the gene expression samples presented in this study were designed for 3 biological replicates (mean ± standard error (SEM), *n* ≥ 3). For the functional enrichment analysis, significantly enriched GO terms, pathways and PPI networks were identified using a *p*-value ≤ 0.05 as the cut off value for statistical significance. GSEA was performed in *Partek*^*™*^ *Flow*^*™*^ software, v11.0 using normalized gene count values. Validation data were plotted and statistically analyzed on GraphPad Prism v9.4.1. software. Bars represented the mean and standard error of the mean. Two-way ANOVA or Unpaired parametric Studenťs *t*-test results were used to compare between groups. Differences were considered statistically significant for *p* values *<* 0.05. *p* values were depicted in graphs as *, **, ***, **** representing *p* ≤ 0.05, *p* ≤ 0.01, *p* ≤ 0.001, and *p* ≤ 0.0001, respectively.

## 3. Results

### 3.1. FUS^P525L^ MNs have slower axonal growth

The P525L mutation in *FUS* has been associated with an early onset and an aggressive form of ALS. An isogenic line carrying this single nucleotide substitution (hereafter FUS^WT^ and FUS^P525L^) was previously generated and characterized. The used small molecule based published protocol allows a high yield of cells that express all major motor neuronal cell markers and demonstrate disease-related phenotypical changes at well-defined time points (Marrone et al., 2018; Naumann et al., 2018; Zimyanin et al., 2023) (Fig. 1A). We quantified FUS protein localization in our line by staining for FUS protein and detected FUS protein mislocalization to the cytoplasm in FUS^P525L^ mutant lines (Fig. 1B-C).

Next, we investigated if axonal outgrowth rates were affected in FUS^P525L^ MNs. We used microfluidic devices with varying lengths of axonal channels, 150 μm, 450 μm and 900 μm, and evaluated the time it takes for the axons enter and cross these channels (Fig. 1D). Both control and FUS^P525L^ MNs^P525L^ were able to cross the majority (*>*90%) of 150 μm channels within 10 days of culture and growth dynamics were comparable. In chambers with 450 μm channels, growth dynamics of FUS^P525L^ MN axons was already visibly slower (18 days for 90% channels crossing in FUS^P525L^ MNs versus 10 days in controls). These differences were even more striking and pronounced for the 900 μm channels, with up to a 10 days delay in ability to cross channels and much lower efficiency or failure to cross all of the channels (35 days for 90% channels in FUS^P525L^ MNs versus 25 days in controls). Our data strongly supports that axonal outgrowth in FUS^P525L^ MNs is strongly compromised and becomes critical especially with requirement for longer distances of growth.

### 3.2. Compartmentalized RNA-seq of iPSC-derived FUS^WT^ and FUS^P525L^ MNs

**Fig. 2.**
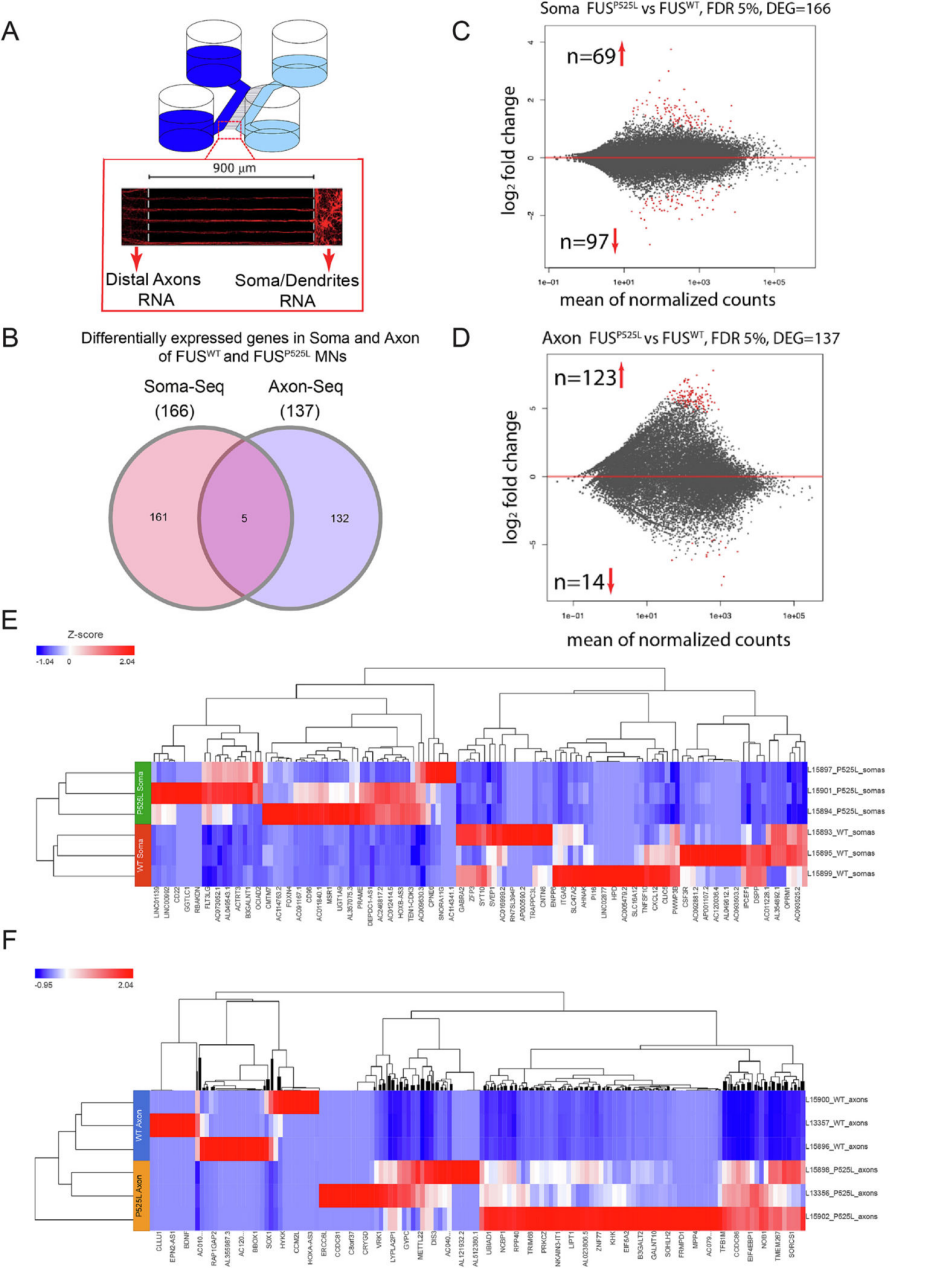
RNA-seq analysis identifies distinct compartment-specific DEGs. (A) Schematic of a microfluidic device and polarized neuron growth used to purify separate RNA from SD and distal axonal compartments. (B) Venn diagram showing number of detected differentially expressed genes in the axons and SDs of the FUS^WT^ and FUS^P525L^ MNs (log2FC ≥ 1 or log2FC ≤ −1, adjusted *p*-value ≤ 0.05, *n* = 3 biological replicas per phenotype). (C-D) MA scatter plots depict significantly changed (adjusted *p*-value ≤ 0.05) DEGs (red dots) with FDR 5% for SD (C) and axons (D) displaying the mean expression (x-axis) and the expression change (y-axis) between conditions. (E-F) The heatmap showing the differential expression of significant (adjusted *p*-value ≤ 0.05, log2FC ≥1) mRNAs in the soma (E) or axon (F) compartment of iPSC derived FUS^WT^ and FUS^P525L^ MNs. Heatmaps were generated in *Partek*™ *Flow*™ software, v11.0 using normalized (separate *Z*-score calculated per gene) expression values (DESeq2) across all samples. Each row in the heatmap represents a sample, and each column represents a mRNA and the color scale at the left of the heatmap represents the Z-score ranging from blue (low expression) to red (high expression), respectively. (For interpretation of the references to color in this figure legend, the reader is referred to the web version of this article.)

We used this cell line to perform a comprehensive subcellular transcriptomic analysis to identify changes that occur in unstressed mutant cells. Additionally, since ALS disease etiology and previous results have shown that some early pre-symptomatic changes occur in axonal compartment, we decided to utilize microfluidic devices to isolate RNA from distal compartments of axons (Fig. 2A). We chose the day 14 in vitro (DIV14) stage of MN maturation as the analysis time point since at this stage axonal morphology is still intact whereas distal axonal organelle trafficking phenotypes can already be detected in the *FUS*-ALS MNs (Fig. 1A) (Pal et al., 2018; Zimyanin et al., 2023). We then used resulting RNA libraries, which were polyA-amplified for axonal samples, and Illumina sequencing to compare the transcriptome of control and FUS^P525L^ mutant MNs.

Our RNA-seq workflow encompassed read quality control (QC), genome mapping, and gene quantification (see Methods). RNA-seq libraries were analyzed from an average depth of ~21–30 million fragments 75 bp single-end reads of good quality (DV200 ≥ 60%) and low amount of rRNA reads (1.1% ± 0.25%). Sequencing reads were then processed to generate normalized counts and the libraries compared using principal component analysis (PCA), indicating that SD samples clearly separated from axon samples (Supplementary Fig. S1A) and is in agreement with Pearson and Spearman’s and unsupervised hierarchical clustering analysis. Pearson and Spearman’s rank correlation coefficient clustering (Supplementary Fig. S1B-E) revealed similar separation of SD from all other samples. Among the whole dataset, the SD samples exhibited an average of 2.38 × 10^4^ (± 264) detectable genes, whereas the axon samples demonstrated remarkably less with an average of 1.3 × 10^4^ (± 3251) genes (Supplementary Table S1). Interestingly, DEGs analysis showed 97 upregulated genes and 69 downregulated genes from SDs (*n* = 166, false discovery rate (FDR) value ≤0.05) and 123 upregulated genes and 14 downregulated genes (*n* = 137) from the axon compartment, selected at the same threshold (Fig. 2B-D) and only minimal overlap of five between both compartments. The unsupervised hierarchical clustering heatmap analysis of the most prevalent DEGs revealed significant differences in expression patterns between the SD and axonal compartments, highlighting those top 50 genes with the highest mean expression strength and highly variable genes across SD and axon samples (FUS^P525L^ versus FUS^WT^ MNs) (Fig. 2E-F, Supplementary Fig. S1F-G). A full list of DEG read values is given in (Supplementary Table S1). In summary, this shows that our experimental pipeline enables RNA-seq of both SD and pure distal axonal preparations of human iPSC-derived MNs and identifies a novel set of distinct compartment-specific transcripts.

### 3.3. Extracellular secretion and extracellular matrix disassembly pathways are important in all distal axonal compartments

To additionally verify the validity of subcellular compartment-specific transcriptome of iPSC-derived MNs, we compared DEGs between SD and axonal compartments of only the FUS^WT^ healthy control samples (Supplementary Table S2). DEG analysis identified 161 genes that were higher expressed in control axons as compared to control SD. Enrichment of the latter genes using the gene ontology (GO; biological process (BP), molecular function (MF), and cellular component (CC) and pathway terms detected inflammatory response, endopeptidase activity, extracellular matrix (ECM), extracellular matrix organization, and activation of metalloproteases pathways/processes, some of which could be important for the correct growth and function of distal axons (Supplementary Fig. S2A-C). DEG analysis also identified a large number of lower expressed transcripts in axons (2482 genes), and these genes were associated with DNA/RNA metabolic processes, nucleic acid catalytic activity, postsynaptic membrane, DNA repair, cell cycle, and *PLK1* pathway, among others, most of which are not usually associated with the axonal functions and were expected to be lower than in the somatodendritic compartment (Supplementary Fig. S2A-C). GO and pathway enrichment results are summarized in Supplementary Table S3. To further identify the functional alterations in specific molecular phenotypes of FUS^WT^ motor axons, we performed gene set enrichment analysis (GSEA) (Subramanian et al., 2005) using the GO term and KEGG pathway databases. Particularly, in axons (versus soma) we found consistent increase of gene sets related to cell-matrix adhesion, tight junction, and WNT signaling, whereas RNA-polymerase based gene transcription, DNA repair, regulation of immune system process, and mitochondrial matrix demonstrated significantly lower expression (Supplementary Fig. S3A–H; Supplementary Table S4).

Interestingly, fewer increased transcripts (25 genes) were detected when comparing axonal and SD transcriptome within FUS^P525L^ MNs. Their enrichment analysis showed some similarity to control enriched terms and pathways, such as secreted peptidase/proteases, peptidoglycan binding, vesicle lumen and ECM disassembly, respectively (Supplementary Fig. S2D-F). On the other hand, lower expressed genes in axons compared to soma (*n* = 332) included a notable prevalence of DEGs linked to cell cycle regulation and DNA damage response. Distinct for FUS^P525L^ MN axon-specific pathways were also glycogen metabolism, WNT signaling and organelle biogenesis (Supplementary Fig. S2D-F). GO and pathway enrichment results are summarized in Supplementary Table S3. Furthermore, GSEA also confirmed increased expression of genes related to mRNA splicing, various types of N-glycan biosynthesis, TCA cycle, and WNT signaling gene sets among others (A–H, Supplementary Table S4). In axons (versus soma), GSEA revealed significantly reduced axonal expression for lipid metabolic process, trans-Golgi network, and MAPK signaling pathway gene sets in FUS^P525L^ MNs, all of which can contribute to the dysregulation of the fine tuning between metabolism and cell survival during development (Supplementary Fig. S4A–H, Supplementary Table S4). Taken together, these data show clear functional differences between transcribed RNA that is present in SD and distal axonal compartments of the same cells. Such differences could be critical for the correct function of axons, highlighting the axonal role of extracellular secretion and ECM that could be perturbed in diseased MNs.

### 3.4. Cross-comparison to published axonal transcriptomes highlights processes characteristic for distal axons

The concept of specialized axonal-specific transcriptome is well accepted for neurons but not well characterized specifically for motor neurons and especially not in the context of ALS. We wanted to compare our novel dataset to the known human and mouse-derived axonal transcripts in motor neurons in order to identify conserved pathways specific for the axons of these cells. Very few studies have analyzed axonal transcriptomes from human and mouse stem cell-derived MNs (Briese et al., 2016; Nijssen et al., 2018). To understand which mRNAs are specific to human motor axons we compared our healthy control axonal-seq datasets with the single published axon datasets derived from iPSC MNs (Nijssen et al., 2018). In this comparison of WT MNs, pathway analysis on the overlapping genes (453 out of 5445 transcripts) revealed significant changes in transcript levels critical for axonal functions including nervous system development, axon guidance and RNA metabolism, including splicing and translation (Supplementary Fig. S2G; Supplementary Table S5). To further investigate if our findings were relevant and conserved even more broadly, we compared our axon datasets with previously published axonal-seq datasets derived from primary mouse MNs and mouse embryonic stem cell (mESC)-derived MNs (Briese et al., 2016; Nijssen et al., 2018). This analysis identified an overlap of 60 common genes, several of which were mainly enriched in Rho GTPase and Notch signaling, as well as somewhat unexpectedly cell cycle and *PLK1* pathways (Supplementary Fig. S2H; Supplementary Table S5). In summary, our cross-comparison analysis of MNs revealed a broadly shared axonal transcriptional signature. It includes known critical axonal related functions, such as RNA metabolism, splicing and translation, as well as ones not usually associated with axons such as *PLK1* and cell cycle pathways. These processes, however could be critical for distal axon maintenance in disease MNs.

### 3.5. Somatodendritic changes upon FUS mutation include WNT signaling, mitochondrial functions and ECM- and synapse-related functions

**Fig. 3.**
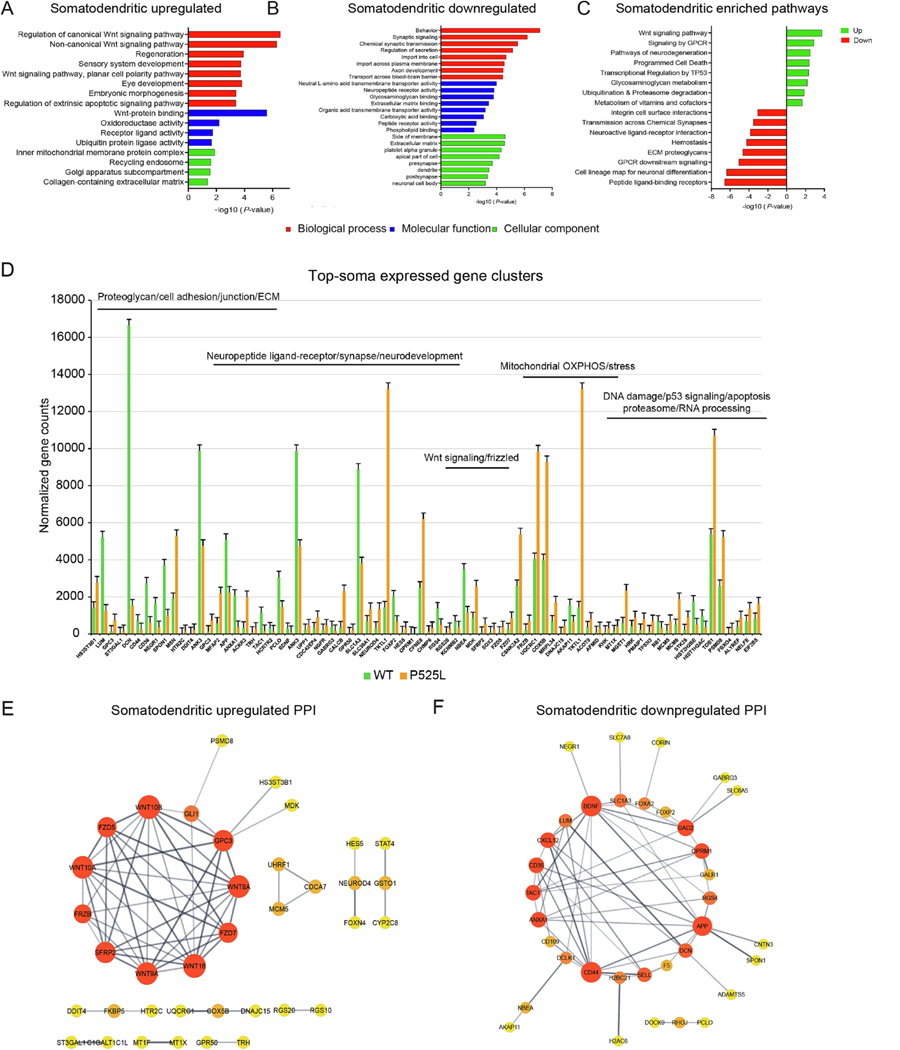
RNA-seq revealed GO, pathway and network analysis of DEGs in SD compartment of FUS^P525L^ MNs. (A-C) Functional analysis of SD-enriched genes identified a variety of (A) upregulated and (B) downregulated GO terms and (C) enriched pathways associated with FUS^P525L^ MNs neurodegeneration (*p* ≤ 0.05). Gene ontology (GO) term enrichment analysis of differential expression genes ranked the most enriched gene group changes in the SD compartment of FUS^P525L^ mutant versus FUS^WT^ healthy control MNs. The enrichment analysis was performed by Metascape and significant terms/pathways were listed with a *p*-value ≤0.05. The GO terms were divided into three categories, including biological process (BP, red), molecular function (MF, blue), and cellular component (CC, green), respectively. See also Supplementary Table S6. (D) Bar graph showing expression levels of the top transcripts in FUS^P525L^ and FUS^WT^ SD related to distinct gene clusters, including ECM, synapse/neuronal functions, WNT signaling, mitochondrial OXPHOS and DNA damage response. All of the genes plotted are significantly enriched in the SD compartment of FUS^P525L^ and FUS^WT^ MNs (*p* ≤ 0.05). *p* values were derived from the DESeq2 differential expression and were adjusted for multiple testing. Data are represented as mean ± SEM. (E-F) STRING analysis of differentially regulated genes involved in protein-protein interactions (PPI) between (E) upregulated and (F) downregulated SD-specific genes showed distinct gene clusters. In the PPI network, each node represents a protein/DEG, and each edge represents an interaction. Node size indicates node degree value and edge color represents STRING enrichment score. Darker/thicker edges mean higher/stronger STRING score. The color of a node depends on its degree values (hubs); the darker the color, the higher the connectivity. Nodes depicted in darker color and with large shape represent their importance to the network. The rank and score of these key genes/clusters were identified with high degree of connectivity within the network (topological parameters), with higher scores in red, middle-range scores in orange, and lower scores in yellow. STRING enrichment analysis for protein-associations of the mapped upregulated and downregulated DEGs in SD datasets showed distinct gene clusters involved in WNT signaling pathway and neuronal- and synapse-related functions. The STRING database was used to establish functional connections among the known and predicted proteins using annotated DEGs as query for SD-specific (FUS^P525L^ versus FUS^WT^ MNs) interaction networks (confidence score ≥ 4) and the generated networks were analyzed as well as visualized using Cytoscape v3.10.1. (For interpretation of the references to color in this figure legend, the reader is referred to the web version of this article.)

For our core analysis we looked at the differences between transcriptomes of FUS^P525L^ and FUS^WT^ MNs. Remarkably, our analysis showed only a small overlap of DEGs between SD and axonal compartments (Fig. 2B), suggesting compartment-specific transcriptional misregulation in the case of *FUS*-ALS. In the SD compartment of FUS^P525L^ MNs upregulated genes were enriched in the regulation of WNT signaling pathway and WNT-protein binding activity in the biological process and molecular function ontology categories, whereas inner mitochondrial membrane and Golgi compartments were identified as major cell compartment (Fig. 3A). Among the DEGs downregulated in FUS^P525L^ MN somas were several key transcripts critical for neuron development and synapse functions and were enriched in the important biological processes including synaptic signaling, chemical synaptic transmission and axon development. In the molecular function and cell component GO categories, enriched terms were associated with neuropeptide receptor activity, ECM binding, and dendrite (Fig. 3B). Furthermore, pathways of upregulated genes were primarily involved in WNT signaling, GPCR signaling and pathways of neurodegeneration (Fig. 3C). The downregulated enriched categories comprised items related to neuronal developmental functions, which included neuronal differentiation, ECM and pathways in peptide ligand-binding receptors (Fig. 3C). The complete GO and pathway analysis results can be found in Supplementary Table S6.

GSEA analysis on SD-enriched transcripts in FUS^P525L^ showed significant upregulation of genes involved in the regulation of apoptotic processes and apoptotic mitochondrial changes, whereas downregulated genes were predominantly involved in nervous system development, proteolysis involved in cellular protein catabolic processes and transGolgi network terms (Supplementary Fig. S5A–H; Supplementary Table S7). Interestingly, significantly changed transcripts in FUS^P525L^ could be clustered into few major processes and included ECM/proteoglycan, neurodevelopmental, WNT signaling, mitochondrial OXPHOS and DNA damage/p53 functions (Fig. 3D). In addition, we used the STRING v12.0 database to map protein–protein interaction (PPI) networks of the mRNA products identified in SD-specific transcripts (148 matched out of 166 identified DEGs) (Dash and Hermann, 2023; Szklarczyk et al., 2015). With the medium confidence level, the upregulated PPI network was constructed with an average node degree of 1.32 and the PPI enrichment *p* = 4.96 × 10^−9^. The network with the upregulated genes (*FZD7*, *FZD5*, *SFRP2*, and *FRZB*) was significantly enriched in WNT signaling pathway (Fig. 3E; Supplementary Table 8), while the downregulated network (average node degree of 2.03 and the PPI enrichment *p* = 1.58 × 10^−11^) was notably enriched in ECM related components (such as proteoglycan, cell adhesion/junction) and synaptic functions (Fig. 3F; Supplementary Table S8). Taken together, these findings indicate that SD of FUS^P525L^ mutant MNs increase the expression of genes regulating neuronal growth/developmental-related functions and WNT signaling, while concomitantly downregulating genes involved with ECM- and synapse-related functions.

### 3.6. Functional enrichment and PPI analysis of axon DEGs centre on PLK1 pathway, mitochondrial gene expression and regulation of inflammation

**Fig. 4.**
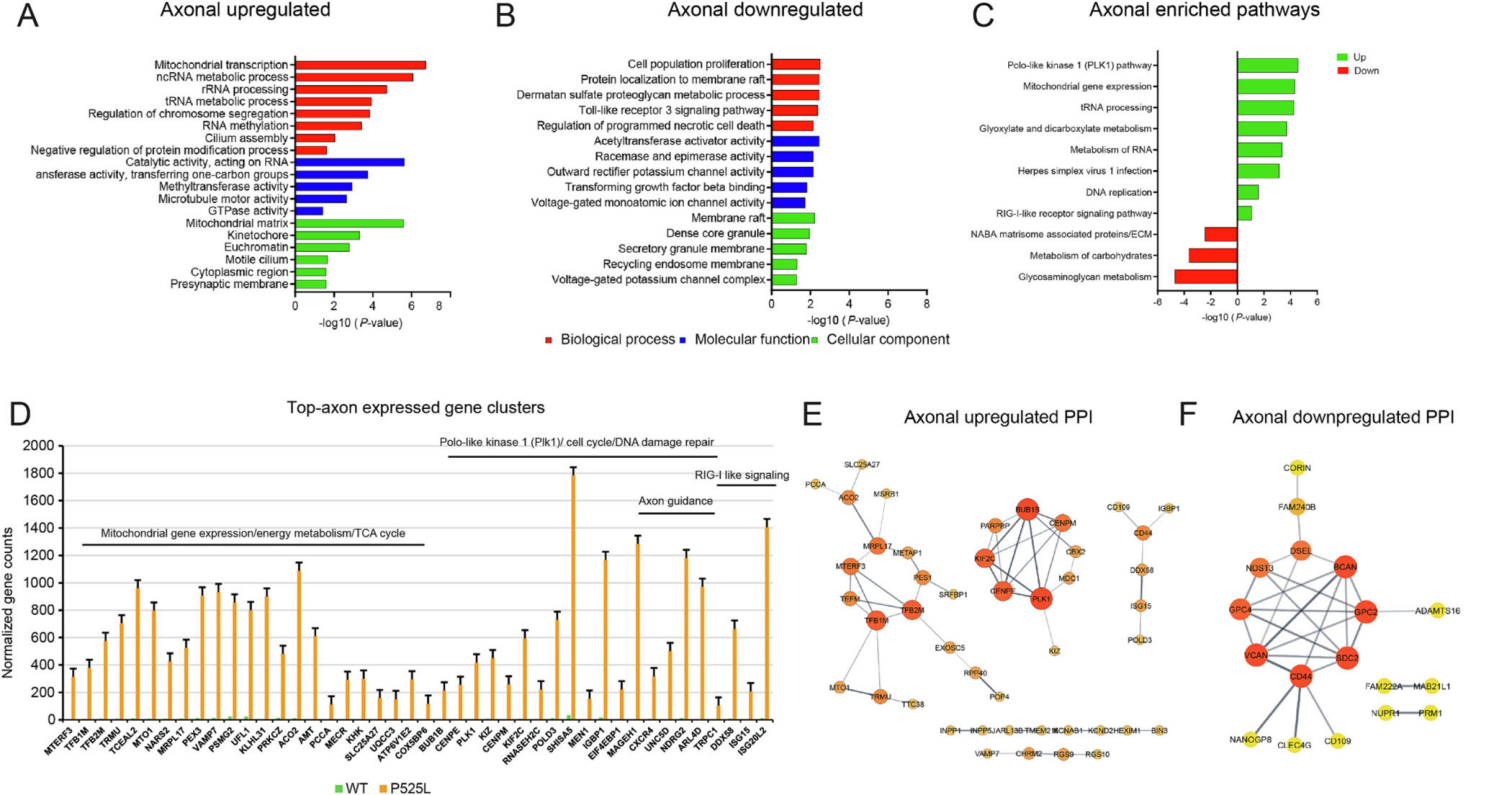
RNA-seq revealed GO, pathway and network analysis of DEGs in axonal compartment of FUS^P525L^ MNs. (A-C) Functional analysis of axonal-enriched genes identified a variety of upregulated (A) and downregulated (B) GO terms and enriched pathways (C) associated with FUS^P525L^ MN neurodegeneration (*p* ≤ 0.05). Gene ontology (GO) term enrichment analysis of differential expression genes ranked the most enriched gene group changes in the axon compartment of FUS^P525L^ mutant versus FUS^WT^ healthy control MNs. The enrichment analysis was performed by Metascape and *p*-value ≤0.05 represents the importance of enrichment. Significantly enriched GO terms in biological process (BP), molecular function (MF), and cellular component (CC) groups were displayed in red, blue and green bars, respectively. See also Supplementary Table S9. (D) Bar graph representation of the top transcripts with the highest expression in axons, a majority of which were connected to functional clusters, including mitochondrial transcription/metabolism, *PLK1* pathway or DNA damage, axon guidance and RIG-I signaling pathways. All the normalized genes plotted are significantly enriched in the axonal compartment of FUS^P525L^ and FUS^WT^ MNs (*p* ≤ 0.05). *p* values were derived from the DESeq2 differential expression and were corrected for multiple testing. (mean ± SEM). (E-F) STRING analysis of differentially regulated genes involved in protein-protein interactions (PPI) between (E) upregulated and (F) downregulated axon-specific genes showed distinct gene clusters. In the PPI network, each node represents a protein/DEG, and each edge represents an interaction. Node size indicates node degree value and edge color represents STRING enrichment score. Darker/thicker edges mean higher/stronger STRING score. The color of a node depends on its degree values (hubs); the darker the color, the higher the connectivity. Nodes depicted in darker color and with large shape represent their importance to the network. The rank and score of these key genes/clusters were identified with high degree of connectivity within the network (topological parameters), with red to yellow-colored nodes represents genes with high to low PPI degree scores. STRING enrichment analysis for protein-associations of the mapped upregulated and downregulated DEGs in axon datasets showed separate gene clusters involved in mitochondrial functions, cell cycle/cytoskeletal organization including *PLK1* pathway, RIG-I signaling pathway and ECM-associated processes. The STRING database was used to establish functional connections among the known and predicted proteins using annotated DEGs as query for axon-specific (FUS^P525L^ versus FUS^WT^ MNs) interaction networks (confidence score ≥ 4) and the generated networks were analyzed as well as visualized using Cytoscape v3.10.1. The STRING database was used to establish functional connections among the known and predicted proteins using annotated DEGs as query for axon- and soma-specific (*FUS*-ALS versus healthy controls) interaction networks (confidence score ≥ 4) and the generated networks were analyzed as well as visualized using Cytoscape v3.10.1. (For interpretation of the references to color in this figure legend, the reader is referred to the web version of this article.)

Changes in the axonal transcriptome of FUS^P525L^ MNs were of special interest in our dataset, as the distal part of the MNs displays some of the earliest visible phenotypes in ALS patients and study model systems. The first obvious difference to the SD compartment was the abundance of DEGs being upregulated in the case of the *FUS* mutant, while more DEGs were downregulated rather than upregulated in the SD compartment (Fig. 2B). Using GO analysis, we observed that these upregulated genes in FUS^P525L^ mutant MN axons were significantly enriched in mitochondrial transcription and RNA metabolic (ncRNA, tRNA, rRNA) processes, RNA-binding, microtubule (MT) motor related functions and mitochondrial matrix components (Fig. 4A), whereas the few downregulated genes were primarily associated with cell proliferation, proteoglycan relevant processes, ion channel activity functions and membrane raft cellular components, implicating aberrant local regulation of distal axonal related processes or functions in the context of FUS^P525L^ mutations (Fig. 4B). Pathway analysis on axon enriched genes revealed that those upregulated in FUS^P525L^ MNs were enriched in the *PLK1* pathway, mitochondrial gene expression and RNA processing pathways. Conversely, genes decreased in expression in FUS^P525L^ MNs were enriched for ECM related functions (Fig. 4C), consistent with shared transcriptional alterations between SD and axon compartments. The complete GO and pathway analysis results can be found in Supplementary Table S9.

GSEA further confirmed that genes involved in mitochondrial RNA/DNA metabolic processes and metabolism as well as DNA damage repair pathways were significantly increased in expression in FUS^P525L^ MN axons, whereas downregulated genes were enriched in several processes/pathways, including regulation of mitochondrial potential, focal adhesion, MAPK and PI3K-Akt signaling pathways (Supplementary Fig. S6A–F; Supplementary Table S7). Many of these dysregulated genes have previously been implicated in axon function and in ALS pathology. Notably, the majority of transcripts with an increased expression in axons were significantly enriched in mitochondrial gene expression/energy metabolism, *PLK1* related cell cycle events/DNA damage and RIG-I signaling as well as axon guidance clusters (Fig. 4D). To verify the functional connectivity of the axon-enriched DEGs, a PPI network was generated based on our upregulated genes (116 matched out of 137 DEGs, average node degree 0.879, PPI enrichment *p* = 7.83 × 10^−2^). Notably, for distal axons we identified three distinct PPI clusters formed by highly connected upregulated protein nodes involved in 1) mitochondrial gene expression/energy metabolism (*TEFM*, *TFB1M*, *TFB2M*, and *MTERF3*, *MRPL17*, *MTO1*, *TRMU*, *ACO2*, *SLC25AA27*), 2) cell cyclebased process/MT cytoskeletal organization (*PLK1*, *KIF2C*, *BUB1B*, *CENPM*, *CENPE*, and *PARPBP*), and 3) RIG-I interferon signaling/inflammation pathways (*DDX58*, *ISG15*, *CD44*, *IGBP1*, *CD109*, and *POLD3*) (Fig. 4E; Supplementary Table S8), potential candidates for specific MN axon (dys-)functions. Subsequently, downregulated genes in the PPI network (average node degree 2.45, PPI enrichment *p* = 2.74 × 10^−5^) were connected to proteoglycan binding and ECM related processes (Fig. 4F; Supplementary Table S8), confirming their essential role in maintaining the normal functions of the motor axons such as axonal growth/regeneration, myelination and cytoskeletal regulation (Barros et al., 2011). Collectively, our findings indicate that axons of FUS^P525L^ MNs are enriched for mRNAs associated with mitochondrial and cell cycle related DNA repair machinery. Upregulation in these pathways may have a critical role in maintaining distal axon energy metabolism and survival/neuronal support as a response to DNA damage pathway activation, an early marker of *FUS*-ALS.

### 3.7. Alternative splicing analysis and comparison with the FUS CLIP-seq profile reveal affected RNA metabolism and splicing pathways, enrichment of mitochondrial and PLK1/mitotic transcripts

FUS is an RNA-binding protein and has been extensively characterized to bind multiple RNA targets and regulating their splicing (Lagier-Tourenne et al., 2012; Orozco et al., 2012; Rogelj et al., 2012). We sought to determine how splicing is affected in the somatodenritic and axonal transcriptome of FUS^P525L^ MNs and whether transcriptomic changes overlap with known mRNA-binding targets of FUS. To analyze alternative splicing we used MAJIQ (version 2.2) (Vaquero-Garcia et al., 2023) and its functions to quantify local variations in splicing and junction usage, comparing all replicates (*n* = 3 biological replicas) across conditions (FUS^WT^ and FUS^P525L^). Detected splice events were classified into intron-retention, alternative 3′ splice site, alternative 5′ splice site and cassette exon, with cassette exon events further subclassified into simple cassette exon, alternative first exon, alternative last exon and complex events. Summary results of this analysis are presented in Supplementary Table 11.

**Fig. 5.**
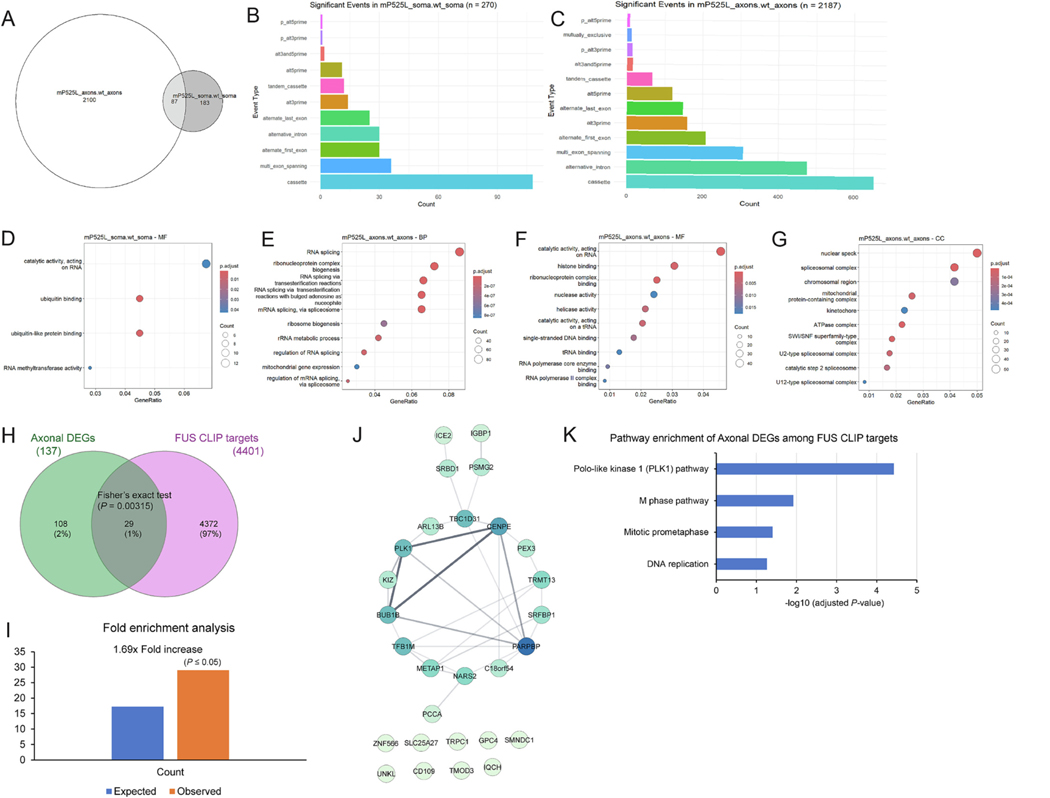
Alternative splicing analysis and comparison with the *FUS* CLIP-seq profile reveal affected RNA metabolism and splicing pathways, enrichment of mitochondrial and *PLK1*/mitotic transcripts. (A) Venn diagrams showing the number of alternative splicing events identified by comparing axonal and somatodendritic compartments between WT and P525L MNs (*n* = 3 biological replicas per group). (B-C) Bar graphs showing the proportional distribution and absolute counts of specific alternative splicing event types detected when comparing the somatodendritic (B) and axonal (C) transcriptomes of P525L versus WT MNs. (D) Gene ontology (GO) enrichment analysis (molecular function, MF) of genes exhibiting differential splicing in the somatodendritic compartment. (E-G)GO enrichment analyses of genes exhibiting differential splicing in the axonal compartment, including biological process (BP) (E), molecular function (MF) (F), and cellular component (CC) (G). (H) Venn diagram showing the overlap of data points between axonal differentially expressed genes (FUS^WT^ and FUS^P525L^) and FUS CLIP targets (*>*1 CLIP site). *p*-value computed using Fisher’s exact test is shown. (I) Bar chart of enrichment showing observed (29 common genes) versus expected (17.2) to visually demonstrate the 1.69× fold increase. Total background size (total number of genes detected by RNA-seq experiment) was used to calculate the significance of enrichment. *FUS* CLIP data are taken from Kügelgen et al., 2025. (J) STRING analysis (*p* ≤ 0.05) of overlapping genes involved in protein-protein interactions (PPI). Disconnected nodes are shown and minimum interaction confidence is set to 0.150. The color of a node depends on its degree values (hubs); the darker the color, the higher the connectivity. Darker/thicker edges mean higher/stronger STRING enrichment score. (K) Bar graph of top enriched pathway terms across lists of input overlapping genes and *p*-value represents the importance of enrichment.

Strikingly, axonal compartments exhibited a substantially higher number of alternative splicing events than somatodendritic compartments, with 2100 differentially spliced mRNA detected in axons compared to only 183 in somatodendritic dataset (Fig. 5A). The proportional distribution of event types was comparable across datasets, with the most common events being cassette and multiexon-spanning events, as well as alternative exon and exon-usage events (Fig. 5B–C). Gene ontology analysis of splicing hits in the somatodendritic compartment identified enrichment for molecular functions related to catalytic activity on RNA, as well as ubiquitin binding and RNA methyltransferase activity (Fig. 5D). A similar analysis of the axonal compartment revealed extensive alterations across multiple processes and pathways, including those linked to splicing itself and ribosomal RNA metabolism (Fig. 5E–G). Consistent with our DEG analysis, we also identified missplicing events affecting mitochondrial gene expression and mitochondrial protein complexes, as well as components of the kinetochore (Fig. 5F–G).

Next, to explore if *FUS* may misregulate axonal hits by binding directly to transcripts encoding them, we utilized *FUS* CLIP data (von Kügelgen et al., 2025) (Fig. 5, Supplementary table 12). The Fisher’s exact test revealed a statistically significant enrichment of *FUS* CLIP sites among axonal DEGs. Specifically, 29 of axonal hits (29 out of 137) contained more than one *FUS* CLIP site, which was a 1.69-fold enrichment over other transcripts (Fig. 5H–I). Unexpectedly, pathway enrichment analysis of these overlapping targets revealed a significant and nearly exclusive enrichment for *PLK1* and DNA replication pathways (Fig. 5J-K). This suggests that axonal misregulation of cellcycle–related machinery may represent a critical pathway affected in FUS^P525L^ MNs. Our data suggest that disruption of cell-division machinery may be a more common pathogenic mechanism in ALS than previously anticipated.

### 3.8. PLK1 expression is upregulated in FUS^P525L^ MNs

**Fig. 6.**
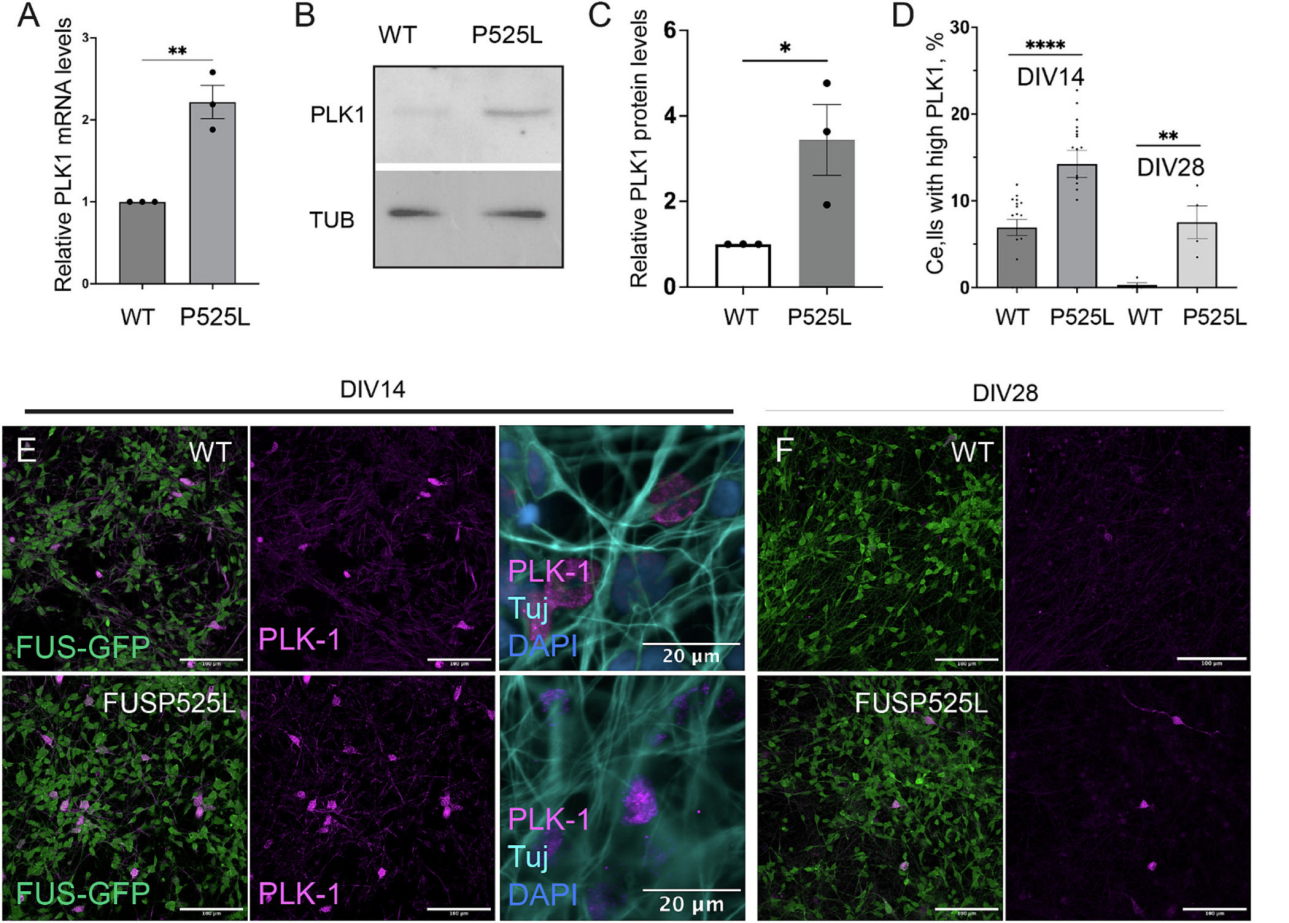
PLK1 is upregulated in FUS^P525L^ MNs. (A) qPCR analysis confirming global relative upregulation of *PLK1* mRNA in FUS^P525L^ MN cultures. mRNA preps from 3 separate mRNA samples were run in triplicates, and were normalized against two different human house-keeping genes (RBS18 and HPRT)) (*p* = 0.0038). (B-C) Representative Western blot (C) and quantification (D) of PLK1 protein levels in whole cell lysates of control and FUS^P525L^ MNs $n_{\exp}^{\text{wt}}=3$, $n_{\exp}^{\text{FUSP}25\text{L}}=3$, biological replicas, *p* = 0.0419. (D-E) Immunostaining for PLK1 shows enrichment of *PLK1* in a subset of differentiated MN at DIV14 is increased in FUS^P525L^ mutant MNs. (E) Quantification of the relative percent of cells showing *PLK1* expression in MNs relative to total number of cells at DIV14 and DIV28 (number of field of view images analyzed $n^{\text{wt}}=17$, $n^{\text{FUSP}25\text{L}}=17$ from 3 independent experiments, p_DIV14_ *<* 0.0001, p_DIV28_ *<* 0.0091) and (F-G) representative images of cells labeled for GFP-FUS, PLK1 and neuronal marker Tuj. Scale bar is 100 μm or 20 μm as labeled in the last panel of F. Error bars are SEM. Significance assessed with unequal variance student *t*-test. * *p* ≤ 0.05, ** *p* ≤ 0.01, *** *p* ≤ 0.001, **** *p* ≤ 0.0001.

Consistent with our results, mutations in familial ALS causing genes such as *TARDBP*, *SOD1*, and *PFN1* were previously associated with upregulation of cell cycle related processes including DNA damage response, and upregulation of *PLK1* particularly in motor neurons (Held et al., 2023; Marques et al., 2024; Naumann et al., 2018; Szewczyk et al., 2025; Waller et al., 2024; Wang et al., 2018; Ziff et al., 2023). Therefore, we decided to test if *PLK1* could be an important key protein and pathway responding to FUS mislocalization in MNs. To confirm that *PLK1* transcript levels are increased in FUS^P525L^ MNs we performed qPCR analysis and found a 2.2 ± 0.2 fold increase in *PLK1* mRNA expression in FUS^P525L^ MNs (Fig. 6A). This result was supported by a 3.4 ± 0.82 -fold-increase in PLK1 protein levels (Fig. 6B, C, Supplementary Fig. S7A). Immunostaining for PLK1 protein showed a relatively weak uniform staining of the MN cells with a small proportion (6.23% ± 0.96) of control cells showing stronger PLK1 signal at DIV14 (Fig. 6D, E). FUS^P525L^ mutant cells had a significantly higher proportion of such cells (14.23% ± 1.55). Most of this high *PLK1* expressing cells were not detected in control cell cultures (0.3%±0.3) by DIV28 but could be still detected in FUS^P525L^ MN cells (7.53%±1.8, *p* = 0.0091) (Fig. 6D, F). These results indicate that *PLK1* activation is part of the phenotype in *FUS*-ALS MNs.

### 3.9. PLK1 activity is increased upon upregulation of DNA damage response

*PLK1* has been shown to possess an increasing number of non-cell cycle related functions (Kumar et al., 2017; Macůrek et al., 2008). For example, *PLK1* was recently reported to be involved in DNA damage repair in a poly(ADP-ribose) dependent manner (Peng et al., 2021; Tsvetkov and Stern, 2005) and is therefore very similar to one of the roles recently associated with *FUS* function (Naumann et al., 2018). We thus wanted to test if *PLK1* upregulation could occur in response to the early upregulation of DNA damage levels in FUS^P525L^ MNs. First, we stressed generic cell culture cells with etoposide to induce DNA damage. Indeed, we found a dose dependent upregulation of *PLK1* upon etoposide treatment in HEK cells in both fluorescence staining as well as western blot (Supplementary Fig. S8 A-D). Excitingly, when investigating MNs, we observed a solid increase in *PLK1* immunofluorescence (IF) signal in the FUS^WT^ MNs upon DNA damage induction, which is however not increased in FUS^P525L^ MNs (Supplementary Fig. S8 E-F). Suggesting that although increase in DNA damage could lead to *PLK1* activation, *FUS* mutant MNs are unable to further respond to such treatment.

### 3.10. FUS^P525L^ MNs do not show cell cycle re-entry but are particularly vulnerable against PLK1 inhibition

**Fig. 7.**
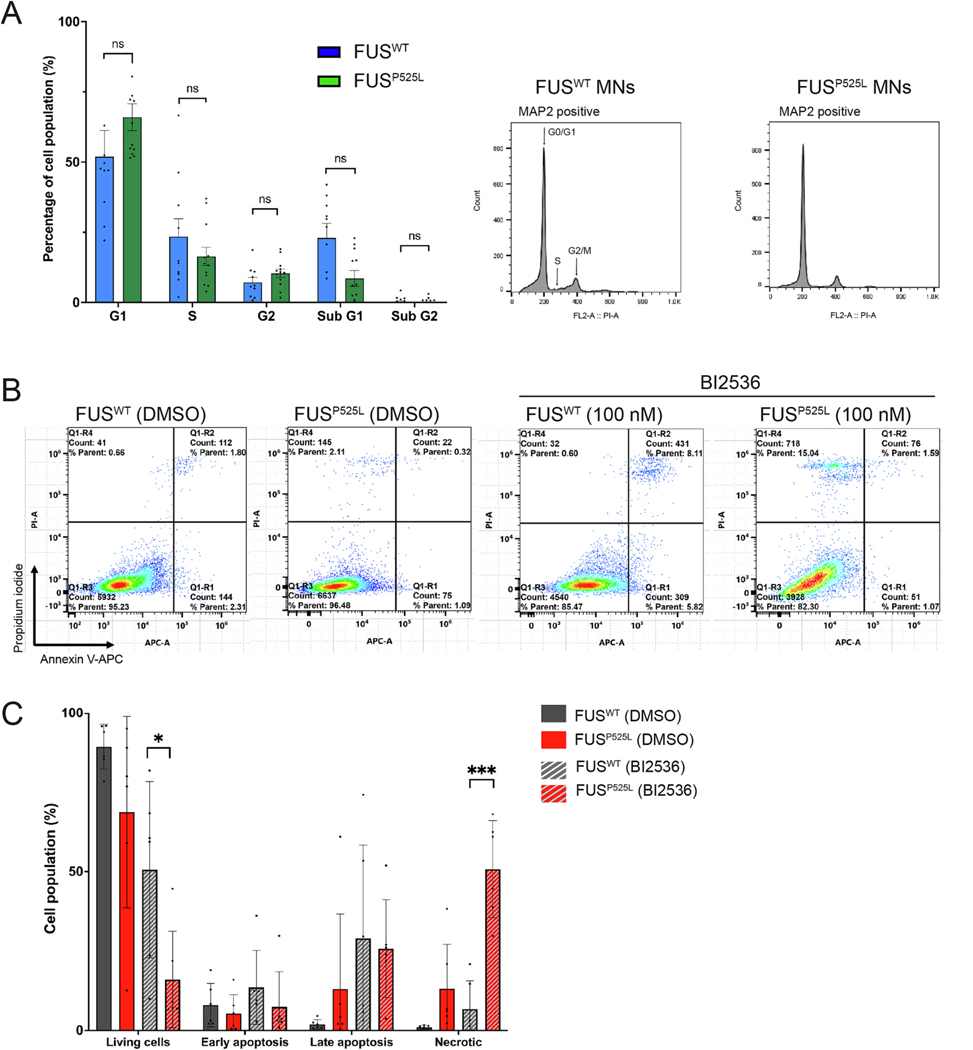
FUS^P525L^ MNs do not show cell cycle re-entry but are vulnerable against *PLK1* inhibition. (A) Percentage of cell cycle stages/distribution calculated from the histogram using the Watson pragmatic algorithm in FLowJo showed no significant effect or change on MAP2 positive cells in FUS^P525L^ compared to FUS^WT^ MNs. Cell cycle profiles (right panel) of untreated MNs (DIV21) by flow cytometry after staining with propidium iodide (PI) showing G0/G1, S and G2/M phases of the cell cycle. Significance was calculated using two-way ANOVA (Tukey’s multiple comparison test). ns = not significant, *n* = 10, mean ± SEM. (B) Flow cytometry analysis results of NeuO+, Annexin V-APC and PI multicolor fluorescence staining showing FUS^WT^ and FUSP^525L^ cell apoptosis/necrosis after DMSO and 100 nM BI2536 treatment (DIV21), as described in Materials and Methods. The percentage of cells in each quadrant is presented as the mean ± SD (standard error of deviation) and the results are representative of at least six independent experiments. (C) Quantitative analysis of apoptotic and necrotic cells using APC Annexin V/PI staining flow cytometry of 72 h in DMSO treated FUS^WT^ control and FUS^P525L^ mutant MNs showed that necrosis process was increased in FUS^P525L^ MNs compared with FUS^WT^ control MNs (D) The number of necrotic cell populations increased significantly in FUS^P525L^ MNs with 100 nM BI2536 treatment (*PLK1* inhibitor) 72 h in culture, whereas BI2536 had no effect on early or late apoptotic cell populations. For each sample, 50,000 events were measured. Dead cells were defined as early apoptotic (Annexin V^+^/PI^−^ ), late apoptotic (Annexin V^+^/PI^+^), and necrotic (Annexin V^−^ /PI^+^). Given are the % numbers of stained cells after treatment. *n* = 6 independent experiments, mean ± SD, p(living cells wt + BI2536 vs P525L + BI2536) = 0.023, p(necrotic cells wt + BI2536 vs P525L + BI2536) = 0.0001. * *p* ≤ 0.05, ** *p* ≤ 0.01, *** *p* ≤ 0.001 in an Unpaired, two-tailed Studenťs *t*-test, asterisks represent *p*-values compared to the controls. All FACS gating strategy figures can be found in supplementary figures.

The canonical function of *PLK1* is required in proliferating cells, during the cell cycle, as well as in cell survival (Iliaki et al., 2021). We first hypothesized that selective upregulation of *PLK1* in the case of *FUS* mutation in MNs is associated with pathological re-entry into the cell cycle, a common feature in a range of neurodegenerative diseases (Nandakumar et al., 2021). To test this hypothesis, we performed cell cycle analysis of neural precursor cells (Supplementary Fig. S9A, B) and MAP2 positive MNs (Supplementary Fig. S10, S11). There was no significant difference in any of the phases of the cell cycle, neither in dividing NPCs nor in post mitotic MNs (Fig. 7A) (Supplementary Figs. S9–S11).

Finally, we investigated whether *PLK1* upregulation might affect cell survival and regulation of cell death pathways like apoptosis or necrosis. For this, we performed multi-color FACS analysis using Annexin V and propidium iodide (PI) labeling in FUS^WT^ and FUS^P525L^ living MNs, which had been labeled live using the neuronal fluorescent dye NeuroFluor™ NeuO (Gating strategy shown in Supplementary Fig. S12, S13). *FUS* mutant MNs did not show significantly increased MNs cell death at DIV 14 of maturation fitting to previous studies (Fig. 7B, C). Finally, we asked whether cell survival is affected in case of *PLK1* inhibition. We first did a titration in proliferating FUS^WT^ and FUS^P525L^ NPCs and finally used 100 nM BI2536 treatment for further analysis, which showed significant block of cell cycle progression in both WT and mutant NPCs (Supplementary Fig. S7A). Treating MNs for 72 h with 100 nM *PLK1* inhibitor BI2536 (or DMSO) reduced survival of MNs and significantly increased apoptotic and necrotic cell death (Fig. 7B, C). This was seen in both FUS^WT^ and FUS^P525L^ mutant MNs, but changes in necrotic cell death rates were significantly increased in case of the FUS^P525L^ mutation.

## 4. Discussion

Accumulation of DNA damage and an early axonopathy with severely impaired distal axonal mitochondria is a hallmark of *FUS*-ALS. However, the reason for this axonopathy as well as the connection to DNA damage is poorly understood. We thus established a compartment-specific RNA-seq approach to identify local changes of transcriptome in axons and compared those to the SD compartments of FUS^WT^ and ALS causing FUS^P525L^ mutant MNs. Such comparison of WT and mutant *FUS*ALS MNs identified fundamental differences between the axonal and SD compartment in general and only a little overlap between detected DEGs between SD and axonal compartments. These differences strongly support the hypothesis that axons contain very distinct sets of RNAs, which in combination with reported local axonal translation mechanisms and different metabolic environment could play a critical role in axonal/synapse function and can pinpoint compartment specific disease mechanisms.

As an example, we detected a general increase in expression of genes that encode ECM components and cell adhesion proteins as a conserved hallmark of axonal compartments in comparison to SD. Several functional studies have reported that upregulation of cell adhesion proteins provides structural/functional connections between ECM molecules and intracellular components and plays a role in axonal growth and survival, neural stem cell behavior, synaptic transmission, and cytoskeletal regulation, implying their importance in axonal plasticity and repair (Previtali et al., 2008). Alterations in these pathways are therefore likely to be significant for ALS pathogenesis and has been reported in at least *C9orf72* and *SOD1* ALS model systems (Milioto et al., 2024; Rossi et al., 2025). In agreement with this, our transcriptome data showed that both SD and axonal transcripts in FUS^P525L^ MNs have a significant downregulation of pathways connected to adhesion, proteoglycan binding and ECM related processes in comparison to FUS^WT^ (Fig. 3B, C, F and 4B, C, F). This was associated with a significant axonal outgrowth phenotype in FUS^P525L^ MNs (Fig. 1D). Thus, our results suggest a potential role of these pathways in the general axonal functions and pathogenesis of *FUS*-ALS and warrants additional investigation.

Our analysis of DEGs in SD compartment of *FUS-*ALS MNs highlighted several other pathways critical for disease development. Upregulation of DNA damage, apoptosis and mitochondrial stress pathways, as well as downregulation of neurodevelopmental processes, synaptic signaling and transmission agrees well with known early *FUS-*ALS phenotypes and cellular defects (Naumann et al., 2018; Wang et al., 2018). Although activation of WNT signaling has been previously implicated in neurodegenerative diseases like Alzheimer’s and Parkinson’s disease (Faraji et al., 2024), only recently it has been also reported in various model systems of ALS and was proposed to modulate oxidative stress, mitochondrial dysfunction, autophagy, and apoptosis (Edens et al., 2017; Hawkins et al., 2022; Soumya et al., 2023). Therefore, our RNA-seq data might help to better understand the role of WNT signaling in ALS and its potential therapeutic implications. Finaly, alternative splicing analysis of our dataset in SD compartment is consistent with the well described role of *FUS* in splicing and we detected a significant effect on pathways affecting RNA metabolism and splicing in both SD and axonal compartment.

Surprisingly, axonal-specific transcriptome analysis detected only a small but well-connected set of enriched DEGs and pathways strongly focused on downregulation of proteoglycan binding and ECM related processes, upregulation of mitochondrial transcription, innate immune response signaling and *PLK1*/cell cycle/DNA damage repair pathways. The latter included the cell cycle and *PLK1* pathway related genes including MT-associated and kinetochore proteins. Comparison of our axonal datasets with other published axonal specific RNA-seq from human and mouse ALS models (Briese et al., 2016; Nijssen et al., 2018) suggests that *PLK1*/cell cycle-related pathways are indeed a commonly activated pathway in axons. Strikingly, both our splicing, and particularly the comparison to known *FUS* CLIPseq targets not only support these results but narrowed them basically down to the latter. It remains unclear why MNs have this baseline expression of *PLK1* even in control non-mitotic cells and what is triggering its upregulation in disease. Based on our experiments we propose that increase in DNA damage levels in *FUS*-ALS MNs could be a potential trigger for *PLK1* activation. Both HEK and control MNs are able to upregulate *PLK1* levels in response to DNA damage stress (Suppl Fig. 8). However, our data would also suggest that once it has been activated in mutant cells, it does not respond to additional increase in DNA damage.

Protective role of *PLK1* in the MNs could be connected to *PLK1* activation in response to DNA damage. *PLK1* is recruited to the double strand breaks induced by laser ablations and such recruitment depends on poly (ADP-) ribose polymerase 1 (PARP-1) and is slowly reversed in PARG-dependent manner (Peng et al., 2021). Interestingly very similar recruitment dynamics and PARP-1/PARG regulation has been also demonstrated for *FUS* and could potentially explain *PLK1* activation in the absence of nuclear FUS (Mastrocola et al., 2013; Naumann et al., 2018). Whether PARP1-dependent *PLK1* recruitment to DNA damage sites could potentially compensate for the lack of *FUS* recruitment or if these mechanisms are independent remains to be tested. Furthermore, the compartment specific upregulation of *PLK1* needs also further investigation. Relevantly, a very recent study reported that *FUS* is involved in mitochondrial DNA repair and *FUS*-ALS mutations disturb mitochondrial DNA damage repair (Kodavati et al., 2024). Of note, mitochondrial damage is mainly found in distal axons in case of *FUS-*ALS. Our axonal data also showed significant disturbance of mitochondrial genes. Thus, it will be important to determine whether *PLK1* is also involved in mtDNA damage repair associated pathways. Finally, Protein Phosphatase 2 A (PP2A) is a phosphatase required for the *PLK1* inactivation during DNA damage response (Wang et al., 2015). Its inhibition or downregulation was found in modifier screens to rescue mitochondrial trafficking defects and neuromuscular junction failure in *FUS* mutant flies and *FUS*-ALS hiPSC-derived MNs (Tziortzouda et al., 2024) and could be also relevant to presented here results.

Activation of *PLK1* can be connected to the coping mechanism of the affected cells. Inhibition of *PLK1* in FUS^P525L^ led to significantly elevated MNs death and diminished their already reduced viability and survival. This is reminiscent to outcomes in many cancer cell model systems, where *PLK1* inhibition compromises survival of cancer cells through various mechanisms including inducing proteasome activity and reducing ubiquitination of proteins (Wang et al., 2024; Zhao et al., 2023). Notably, disturbance of protein homeostasis is a key hallmark and perhaps determinant of numerous neurodegenerative disorders, including ALS (Brauer et al., 2018; Szewczyk et al., 2023). Our data does not specifically determine if *PLK1* has a direct role in downregulating these cellular processes, and whether specific cell death pathways were involved. However, recent evidence strongly implicated ferroptotic cell death upregulation in several ALS models including *FUS*-ALS, which is a form of necrotic cell death (Ismail et al., 2024; Wang et al., 2022). Along this line, *PLK1* function has been shown to inhibit ferroptosis in esophageal squamous cell carcinoma through activity of pentose phosphate pathway and regulation of NADPH and glutathione levels (Zhao et al., 2023). Potential role of ferroptosis regulation remains to be investigated in the context of this study.

Additional mechanism for downstream axonal degeneration mechanisms would involve potential role of regulating cellular innate immune response mechanisms in ALS (McCombe and Henderson, 2011). Such hypothesis is supported by the fact that one of the few identified PPI networks in our distal axonal analysis maps connected to the immune response reporter RIG-I/interferon/inflammation and Herpes simplex virus 1 infection pathways. The latter would also fit the hypothesis of retrotransposon and virus reactivation as potential contributing mechanisms of ALS (Dash et al., 2020; Tam et al., 2019). In addition to the immune-related enriched terms from the PPI analysis, we also identified a network related to activation of mitochondrial transcription/metabolism pathways in FUS^P525L^ mutant motor axons, as compared to the healthy controls, and GSEA further showed increased expression across the mitochondrial functional gene set. This finding was intriguing given that prior studies in MNs and postmortem tissues have demonstrated crucial function of *FUS* in maintaining mitochondrial DNA/RNA integrity following overexpression of *FUS* and through its involvement in repair mechanisms (Deng et al., 2015; Guo et al., 2017; Kodavati et al., 2024; Naumann et al., 2018; Tsai et al., 2020). Above observations were further validated in our parallel study that detected increased mitochondrial dsRNA, upregulation of RIG-I and interferon-stimulated genes in *FUS-*ALS MNs (Naumann et al., 2024). It will be critical to further address protective mechanisms underlying mitochondrial function and transcriptional fidelity in FUS^P525L^ MN axons, which are highly reliant on mitochondria to meet their energy demands and could be affected by upregulated immune response pathways.

Although our FACS sorting results failed to detect it, we cannot categorically rule out some form of aberrant cell-cycle re-activation in ALS. However, the hypothesis we want to propose, and which we believe is supported by this work, is that the recurring detection of increased cell-cycle-related transcripts in our results, and their significant enrichment in the overlap with known *FUS* CLIP-seq targets, could represent their aberrant activation and axonal localization in response to some of the mechanisms described above, and that cell-division machinery may be a more common pathogenic mechanism in ALS than previously anticipated.

In summary, our results provide a powerful resource to better understand compartment specific changes in neuronal health and diseases. It further highlights shared axonal transcriptional signature across species, including known critical axonal related functions, such as RNA metabolism, splicing and translation, as well as ones not usually associated with axons such as *PLK1* and cell cycle pathways. Of interest is that the latter are selectively expressed by spinal MNs (Held et al., 2023; Szewczyk et al., 2025). In *FUS*-ALS, ECM related pathways were downregulated in SD and axon compartment, being most likely responsible for the observed impairment of axonal outgrowth in *FUS*ALS MNs. Finally, we propose that the additional *PLK1* pathway activation in *FUS*-ALS is a response to the increased DNA damage observed in these cells and could provide a protective mechanism for the survival of these affected cells. *PLK1* activation could thus provide a potential link between increased DNA damage, activation of inflammatory pathways, distal mitochondrial axonal phenotypes and compromised cell survival in *FUS*-ALS MNs, which deserves further systematic functional studies. It becomes evident that some prominent underlying symptoms and biomarkers are present in patients from early on and our focus on misregulated pathways we present here would be critical for our potential ability to detect, modulate and prevent this debilitating disease.

Supplementary data to this article can be found online at https://doi.org/10.1016/j.nbd.2026.107268.

## Supplementary Material

MMC14

MMC15

MMC16

MMC17

MMC18

MMC19

MMC20

MMC21

MMC22

MMC23

MMC24

MMC25

MMC26

MMC6

MMC1

MMC11

MMC9

MMC10

MMC3

MMC12

MMC5

MMC4

MMC8

MMC2

MMC7

MMC13

## Data Availability

Data are deposited at NCBI/GEO under the accession number GSE276214.
